# Deciphering the gut-liver axis in MASLD: from dysbiosis-driven pathogenesis to precision microbiome therapeutics

**DOI:** 10.3389/fmicb.2026.1971785

**Published:** 2026-09-29

**Authors:** Wenxin Liu, Yi Lan, Xia Chen, Hui Wei, Tianjiao Zhang, Yuping Wang

**Affiliations:** 1 The First Clinical Medical College, Lanzhou University, Lanzhou, China; 2 Department of Gastroenterology, The First Hospital of Lanzhou University, Lanzhou, China; 3 Gansu Province Clinical Research Center for Digestive Diseases, The First Hospital of Lanzhou University, Lanzhou, China; 4 Department of Nephrology, The First Hospital of Lanzhou University, Lanzhou, China

**Keywords:** bile acid metabolism, fecal microbiota transplantation, gut microbiota, gut-liver axis, metabolic dysfunction-associated steatotic liver disease, therapeutic strategies

## Abstract

Metabolic dysfunction-associated steatotic liver disease (MASLD) has emerged as the most prevalent chronic liver disorder worldwide, characterized by a multifactorial pathogenesis involving genetic predisposition, metabolic disturbances, environmental influences, and gut microbiota interactions. In recent years, the gut microbiota has garnered significant attention for its pivotal role in MASLD development and progression through the gut-liver axis. This review comprehensively summarizes current understanding of the molecular mechanisms by which gut microbiota and their metabolites contribute to MASLD pathogenesis, including intestinal barrier dysfunction, bile acid metabolism dysregulation, abnormalities in short-chain fatty acid and tryptophan metabolism, oxidative stress, and mitochondrial impairment. Furthermore, we comprehensively discuss advances in therapeutic strategies targeting gut microbiota modulation, such as probiotics/prebiotics, fecal microbiota transplantation, dietary interventions, bioactive compounds from traditional Chinese medicine, and surgical approaches. By integrating these insights, this review aims to provide novel perspectives for precise prevention and treatment of MASLD, highlighting the gut microbiota as a promising target for future clinical management.

## Graphical abstract

**Figure:**
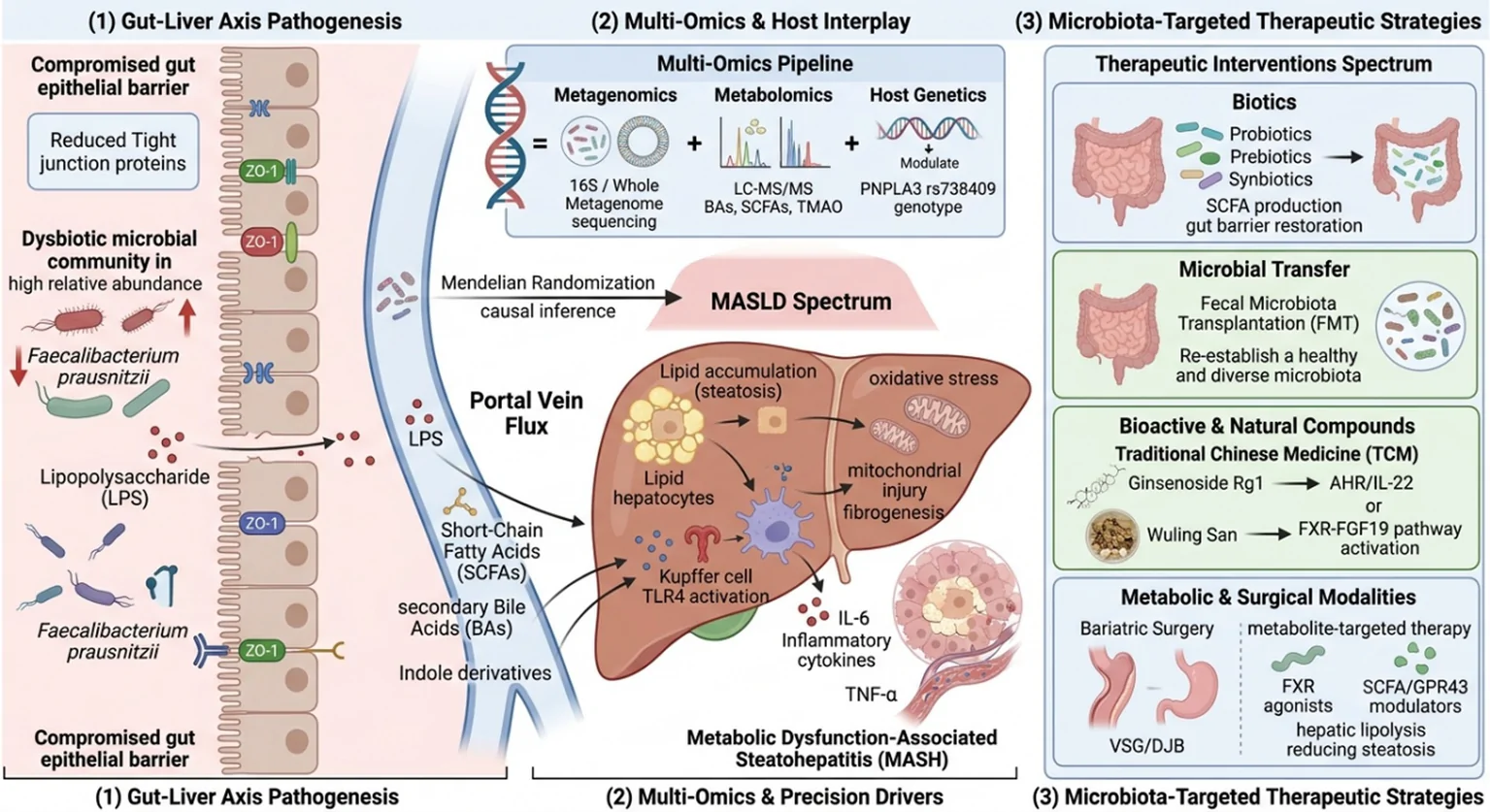

## 1 Introduction

Metabolic dysfunction-associated steatotic liver disease (MASLD) has emerged as a predominant global health challenge, characterized by abnormal lipid accumulation in hepatocytes accompanied by metabolic dysregulation. This clinical-pathological syndrome encompasses a broad disease spectrum ranging from simple hepatic steatosis to metabolic dysfunction-associated steatohepatitis (MASH), advancing to fibrosis, cirrhosis, and ultimately hepatocellular carcinoma (HCC) (Huang et al., 2025). MASLD affects over one-third of adults worldwide and is tightly linked to prevalent metabolic conditions such as obesity, insulin resistance, and type 2 diabetes mellitus (T2DM), which have surged in parallel with the global obesity epidemic. The redefinition of this disease entity from the former non-alcoholic fatty liver disease (NAFLD) to MASLD reflects a paradigm shift emphasizing the central role of metabolic dysfunction and cardiometabolic risk factors in its pathogenesis, thereby improving clinical recognition and reducing stigmatization associated with the term “fatty” liver (Yoon and Jun, 2023). Accordingly, the term MASLD is used throughout this review; NAFLD is retained only when referring specifically to studies conducted under the former nomenclature or when reproducing original article titles. The clinical importance of MASLD is underscored by its association with increased liver-related morbidity and mortality. Inflammatory processes, mediated by diverse cytokines and immune cells, drive progression from simple steatosis to MASH and fibrosis. Cytokines such as tumor necrosis factor-alpha (TNF-α), interleukins (IL-6, IL-1β), and transforming growth factor-beta (TGF-β) orchestrate hepatic inflammation and fibrogenesis, representing potential biomarkers and therapeutic targets (Vachliotis and Polyzos, 2025). Moreover, mitochondrial dysfunction and impaired quality control mechanisms, including defective mitophagy, contribute to hepatocellular injury and metabolic derangements characteristic of MASLD. Recent studies have identified key molecular regulators such as RNF128, which stabilizes stearoyl-CoA desaturase 1 (SCD1) to promote lipid accumulation, and USP2, which enhances peroxisome proliferator-activated receptor gamma (PPARγ) activity, further elucidating the intricate intracellular pathways involved. These insights provide a foundation for developing targeted pharmacotherapies aimed at modulating lipid metabolism, inflammation, and fibrosis.

The gut microbiota, as the largest and most complex microbial ecosystem in the human body, has gained increasing recognition for its pivotal role in MASLD pathogenesis through the gut-liver axis. Dysbiosis, characterized by altered microbial composition and function, influences hepatic lipid metabolism, immune responses, and fibrogenesis via microbial components and metabolites. Structural components such as lipopolysaccharide (LPS) can trigger hepatic inflammation, while microbial metabolites including short-chain fatty acids (SCFAs), bile acids, tryptophan derivatives, and trimethylamine N-oxide (TMAO) modulate host metabolic and immune pathways. For instance, butyrate-producing bacteria such as *Faecalibacterium prausnitzii* and Eubacterium are often depleted in MASLD patients, correlating with disease severity and metabolic comorbidities (González-González et al., 2026). Beyond the bacteriome, non-bacterial components of the intestinal microbiome are increasingly recognized as relevant to MASLD. Gut fungi, particularly *Saccharomyces cerevisiae*, have been implicated in MASLD through modulation of tryptophan-derived metabolites such as 5-hydroxyindoleacetic acid (5-HIAA) (Qiao et al., 2026), while recent human mycobiome studies have identified disease-associated fungal taxa and altered fungal-bacterial-metabolite networks (Zheng et al., 2025). The intestinal virome, which is dominated by bacteriophages, may further reshape bacterial community structure and metabolic function; altered viral diversity, phage composition, and phage-host interactions have been associated with NAFLD/MASLD and disease severity (Lang et al., 2020; Wang, L. et al., 2025; Zhou, X. et al., 2026). These observations suggest that the gut-liver axis should be considered as a multi-kingdom ecological network involving bacteria, fungi, viruses, and their metabolic interactions rather than as a bacterium-centered system alone.

Advances in multi-omics technologies—such as metagenomics, metabolomics, and 16S rRNA sequencing—have facilitated comprehensive characterization of gut microbiota alterations and their metabolic outputs in MASLD. These approaches, combined with fecal microbiota transplantation (FMT) experiments, have begun to unravel causal relationships and molecular mechanisms linking dysbiosis to liver disease progression. However, clinical trials of FMT in MASLD have yielded mixed results, potentially due to patient heterogeneity and baseline microbiota diversity, underscoring the need for personalized microbiota-targeted interventions (Groenewegen et al., 2025). Furthermore, gut microbiota-derived metabolites such as indole-3-propionic acid (IPA) and indole-3-acetic acid (IAA) have demonstrated hepatoprotective effects by modulating inflammatory signaling and lipid metabolism, suggesting novel therapeutic avenues (Min et al., 2024).

Therapeutic strategies targeting the gut microbiota and metabolic dysfunction in MASLD are rapidly evolving. Lifestyle modifications including dietary interventions, physical exercise, and weight loss remain foundational, with time-restricted eating and resistant starch supplementation showing promise in improving hepatic steatosis and metabolic parameters, partly through gut microbiota modulation. Pharmacological treatments have advanced with the recent FDA approvals of resmetirom, a thyroid hormone receptor β-selective agonist, and semaglutide, a glucagon-like peptide-1 receptor agonist, for MASH with fibrosis, marking a new era in MASLD management. Antiobesity medications such as tirzepatide and retatrutide also demonstrate significant efficacy in reducing hepatic fat and resolving steatohepatitis. Additionally, natural products like myricetin and alisol B have shown potential in preclinical models by enhancing mitochondrial quality control and restoring metabolic homeostasis, respectively. Bariatric surgery offers another effective intervention for MASLD patients with obesity, improving liver histology beyond weight loss alone (Liu et al., 2025). Collectively, these interventions underscore the importance of integrated approaches addressing metabolic, microbial, and inflammatory pathways. Importantly, emerging evidence suggests that clinically relevant pharmacotherapies may interact bidirectionally with the gut microbiome and bile acid metabolism, potentially influencing both their downstream metabolic effects and interindividual treatment responses, as discussed in Section 4.6 (Sun et al., 2024; Zhang, Y. H. et al., 2025; Klemets et al., 2026; Hu et al., 2025).

In summary, MASLD represents a complex metabolic liver disorder with multifactorial etiology involving metabolic dysfunction, immune dysregulation, and gut microbiota alterations. The gut-liver axis emerges as a critical interface mediating disease progression and therapeutic response. Recent advances in molecular pathogenesis, microbiome research, and clinical therapeutics provide a robust framework for developing personalized prevention and treatment strategies. This review aims to critically and comprehensively synthesize current knowledge on the role of gut microbiota in MASLD pathogenesis and therapy, highlighting emerging mechanistic insights and translational opportunities to inform individualized patient care.

## 2 Gut microbiota and MASLD: correlation and epidemiological evidence

### 2.1 Characteristic alterations in gut microbiota composition in MASLD patients

Extensive cross-sectional studies and meta-analyses have consistently demonstrated significant dysbiosis of the gut microbiota in patients with metabolic dysfunction-associated steatotic liver disease (MASLD). A hallmark of this dysbiosis is the reduction in overall microbial diversity, which is a critical indicator of gut ecosystem health. Specifically, beneficial bacterial genera such as Faecalibacterium and Bifidobacterium are markedly decreased in MASLD patients, while potentially pathogenic taxa including *Escherichia coli* and certain Bacteroides species show increased abundance. These compositional shifts suggest a disrupted microbial balance that may contribute to disease pathogenesis. Notably, pediatric studies have linked increased *Ruminococcus torques* abundance to higher deoxycholic acid levels and greater liver stiffness, suggesting a potential microbiota–bile acid association with disease severity (Jiang et al., 2026). A recent adult case–control study using shotgun metagenomic sequencing likewise reported a higher abundance of *R. torques* in adults with MASLD than in healthy controls, although the pediatric DCA–liver stiffness association was not directly reproduced (Williams et al., 2025). These findings suggest that *R. torques* may represent an age-spanning candidate microbial marker, while the functional consequences and strength of its association with disease severity may differ between pediatric and adult MASLD (Jiang et al., 2026; Williams et al., 2025). Furthermore, interactions between gut microbiota composition and host genetic susceptibility have been elucidated; for example, carriers of the PNPLA3 rs738409 CC/CG genotypes exhibit decreased levels of Blautia and Ruminococcaceae in the MASLD state, whereas such associations are absent in GG genotype carriers. This gene-microbiota interplay underscores the complexity of MASLD pathogenesis and highlights the importance of personalized approaches. Mendelian randomization studies have further provided genetic evidence supporting a causal relationship between specific gut microbial taxa and MASLD, identifying 11 bacterial groups with potential causal links to the disease. These convergent observational and genetic data provide a basis for evaluating selected microbial taxa as candidate biomarkers or intervention targets, although their reproducibility and causal relevance across independent populations require further validation (Lau et al., 2025; Saeed et al., 2025; Guo et al., 2026).

### 2.2 Loss of gut microbiota diversity and disease progression

A consistent and critical feature of gut microbiota dysbiosis in MASLD is the loss of α-diversity, reflecting diminished microbial richness and evenness. Alterations in microbial diversity have been reported in both adult and pediatric MASLD, but the corresponding microbial signatures are not necessarily interchangeable across age groups. In pediatric MASLD, shotgun metagenomic meta-analysis has demonstrated significant alterations in both α- and β-diversity across obesity, MASLD, and MASH, with changes in species such as *Faecalibacterium prausnitzii* and *Prevotella copri* (Zöggeler et al., 2025). Notably, microbiome-based machine-learning models could distinguish pediatric from adult MASH with an AUROC of 0.97, indicating substantial age-dependent differences in microbial community structure (Zöggeler et al., 2025). The Gut Microbiota Health Index has also been reported to decrease in pediatric MASLD and to associate with biochemical or inflammatory indices of liver injury (Jiang et al., 2026). However, because these observations were derived from pediatric populations, equivalent performance should not be assumed in adults without independent validation. Adult MASLD cohorts have identified distinct severity-associated microbial patterns, including enrichment of Enterococcus, Granulicatella, and Morganellaceae in MASH and associations between other microbial configurations and severe steatosis (Borges-Canha et al., 2025; Schwenger et al., 2025). Thus, age should be considered an important source of microbiome heterogeneity when microbial biomarkers are interpreted or translated into clinical stratification. Mechanistically, sustained loss of microbial diversity compromises the stability and functional redundancy of the gut ecosystem, weakening its resilience against colonization by pathogenic bacteria. This disruption facilitates a vicious cycle of dysbiosis, intestinal barrier impairment, and exacerbated hepatic inflammation. The compromised gut barrier allows translocation of microbial products such as lipopolysaccharide (LPS) into the portal circulation, triggering hepatic immune activation and promoting disease progression from simple steatosis to steatohepatitis and fibrosis. Thus, maintaining or restoring microbial diversity represents a critical therapeutic goal to halt or reverse MASLD progression (Lau et al., 2025; Benedé-Ubieto et al., 2024).

### 2.3 Dynamic evolution of gut microbiota across different disease stages

The gut microbiota undergoes dynamic compositional and functional changes as MASLD progresses from simple steatosis to metabolic dysfunction-associated steatohepatitis (MASH) and eventually to cirrhosis. During the MASH stage, there is an enrichment of pro-inflammatory bacterial genera alongside a further decline in beneficial metabolically active taxa, reflecting an increasingly dysbiotic state that exacerbates hepatic inflammation and injury. At the phylum level, significant alterations in the ratio of Bacteroidetes to Firmicutes have been observed, with a notable loss of butyrate-producing Firmicutes members correlating with advancing liver fibrosis. Butyrate, a short-chain fatty acid, is essential for maintaining intestinal barrier integrity and modulating immune responses; its depletion contributes to barrier dysfunction and systemic inflammation. Importantly, several microbiota-based classification models have been developed to distinguish advanced stages of steatotic liver disease from milder disease. In a prospective study conducted under the former NAFLD nomenclature, Loomba et al. applied whole-genome shotgun metagenomic sequencing to 86 patients with biopsy-characterized disease and developed a Random Forest classifier incorporating 37 bacterial species together with Shannon diversity, age, and body mass index. The model distinguished mild-to-moderate fibrosis (F0–F2) from advanced fibrosis (F3–F4) with an area under the receiver operating characteristic curve (AUROC) of 0.936 (Loomba et al., 2017). Caussy et al. subsequently developed a 30-feature model comprising 27 bacterial features together with age, sex, and body mass index. This model achieved an AUROC of 0.92 for detecting cirrhosis in the derivation cohort and retained an AUROC of 0.87 in an independent validation cohort of first-degree relatives, suggesting that fecal microbial signatures may contain information relevant to advanced disease stratification (Caussy et al., 2019).

However, the performance of microbiota-based classifiers has not been uniformly reproducible across populations and analytical platforms. In an independent cohort, Lang et al. attempted to approximate and validate the previously reported metagenomic signature using 16S rRNA sequencing; the resulting model achieved an AUROC of only 0.71 for advanced fibrosis (Lang et al., 2020). A newly trained model combining bacterial taxa with clinical variables achieved an AUROC of 0.87, but this was only marginally higher than a model based on clinical variables alone (AUROC 0.85) and was inferior to transient elastography (AUROC 0.93, 28). These findings indicate that the apparent diagnostic performance of microbiome-based classifiers may depend on cohort composition, geographic and dietary background, sequencing methodology, taxonomic resolution, and the clinical variables incorporated into the model. Therefore, although microbial signatures provide a promising framework for non-invasive MASLD stratification, their incremental value over established fibrosis assessment tools and their generalizability across independent populations require further prospective validation before routine clinical application (Lau et al., 2025; Loomba et al., 2017; Caussy et al., 2019; Lang et al., 2020).

### 2.4 Beyond the bacteriome: gut mycobiome and virome in MASLD

Although bacterial dysbiosis remains the most extensively studied component of the gut microbiome in MASLD, increasing evidence indicates that fungi and viruses constitute additional layers of the gut-liver axis. The intestinal mycobiome has received particular attention because fungi can influence host metabolism both directly through fungal-derived metabolites and indirectly through cross-kingdom interactions with bacterial communities. Recent work identified a potentially protective *Saccharomyces cerevisiae*–5-HIAA–AHR axis, in which *S. cerevisiae* promoted intestinal 5-HIAA production and enhanced hepatic fatty acid oxidation (Qiao et al., 2026). However, fungal alterations appear to be species- and population-dependent rather than reflecting a uniform change in total fungal diversity. In a human metagenomic analysis of 90 patients classified according to the former NAFLD framework and 90 matched controls, overall fungal diversity and global community structure did not differ significantly between groups, whereas specific taxa, including Pseudopithomyces, Mucor, Aspergillus, and Ascochyta species, were enriched in the disease group and showed associations with serum metabolites (Zheng et al., 2025). Notably, *S. cerevisiae* abundance was not significantly altered in this cohort, illustrating that individual fungal signatures may not be reproducible across populations or analytical platforms (Zheng et al., 2025). These observations caution against categorizing the mycobiome simply as protective or pathogenic and emphasize the need for species- and strain-resolved analyses of fungal function.

The gut virome represents another understudied component of MASLD, with bacteriophages accounting for a substantial proportion of intestinal viral communities. By infecting bacterial hosts, bacteriophages can regulate bacterial abundance through lytic predation, modify bacterial fitness through lysogeny and horizontal gene transfer, and influence microbial metabolic functions. In a fecal virome study conducted under the former NAFLD nomenclature, advanced disease activity and fibrosis were associated with reduced viral diversity and a lower relative proportion of bacteriophages, and incorporation of viral diversity improved models for identifying severe disease (Lang et al., 2020). More recent human data, however, suggest that global viral diversity may not be uniformly altered. A metagenomic study comparing 90 patients classified using the former NAFLD terminology with 90 controls found no significant difference in overall viral diversity but identified extensive changes in specific viral operational taxonomic units and their predicted bacterial hosts. Disease-associated phages preferentially targeted Bacteroides, whereas phages associated with Faecalibacterium, Oscillibacter, and Prevotella were more prominent in controls (Wang, L. et al., 2025). Thus, the biologically relevant virome signal may reside more in phage composition, host range, and functional capacity than in global diversity alone.

Importantly, recent multi-kingdom analyses support substantial ecological interactions among bacteria, fungi, bacteriophages, and microbial metabolites. In a cohort integrating metagenomics, fungal ITS2 sequencing, fecal metabolomics, and clinical profiling, MASLD was associated with disruption of phage-bacteria-metabolite networks, including depletion of *Faecalibacterium prausnitzii* and its associated bacteriophages and reduced predicted lytic control of the MASLD-enriched species *Ruminococcus gnavus* (Zhou, X. et al., 2026). These alterations were accompanied by changes in microbial functional genes and secondary bile acid profiles, suggesting that bacteriophages may influence the gut-liver axis indirectly by restructuring bacterial communities and their metabolic outputs (Zhou, X. et al., 2026). Nevertheless, current mycobiome and virome evidence remains predominantly associative, and direct causal relationships between specific fungi or phages and human MASLD progression have not yet been established. Future studies should therefore integrate strain-resolved bacterial metagenomics, dedicated virome sequencing, fungal profiling, and metabolomics in longitudinal cohorts, followed by experimental validation of candidate cross-kingdom interactions before fungal- or phage-targeted therapies are considered for clinical translation (Figure 1) (Zheng et al., 2025; Wang, L. et al., 2025; Zhou, X. et al., 2026).

**Figure 1:**
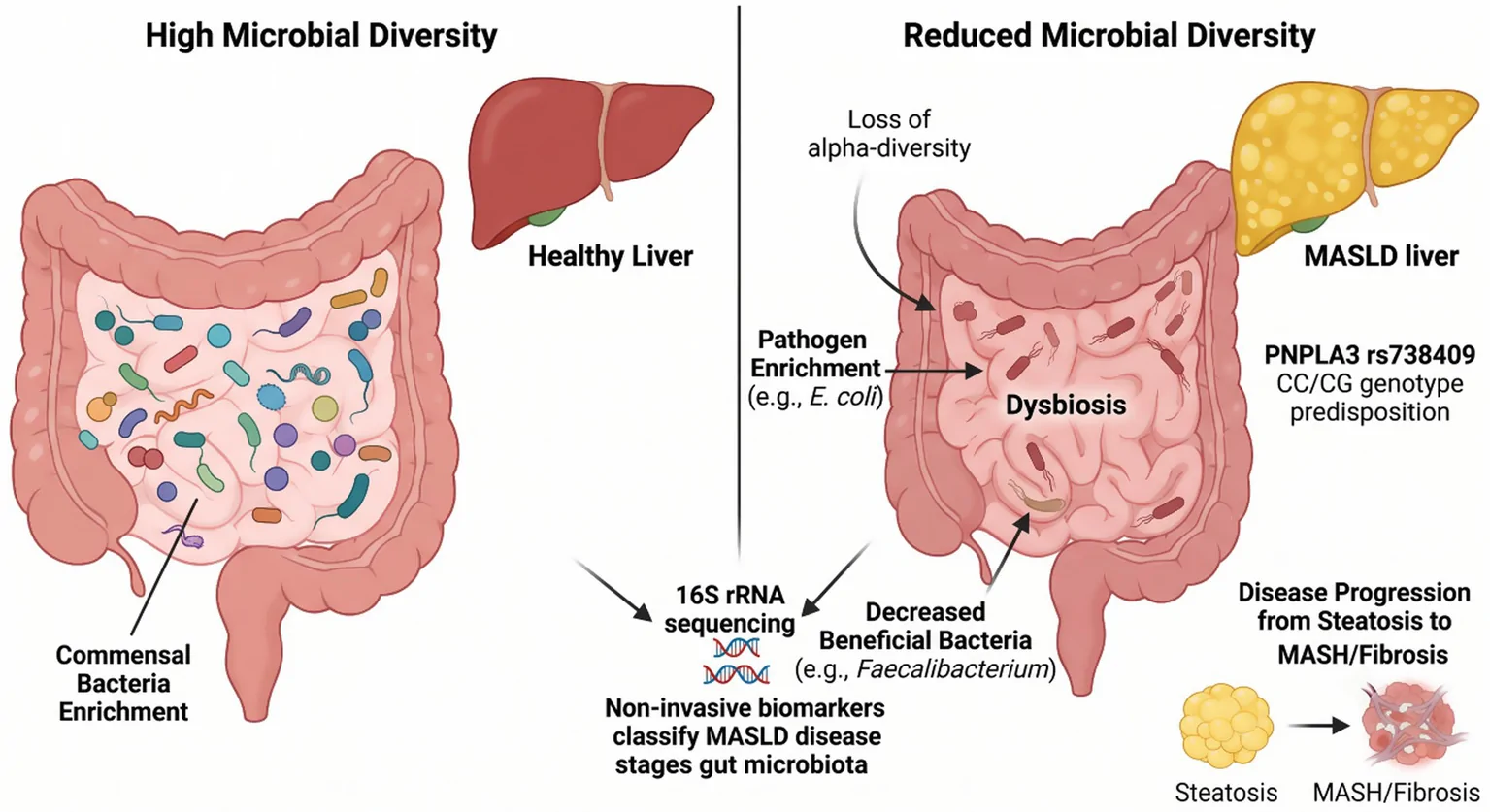
Challenges and opportunities in gut-liver axis research. The schematic outlines three primary challenges in studying the gut-liver axis and proposes innovative solutions. First, current indirect assessments (fecal/blood sampling) overlook real-time liver flux, suggesting direct portal vein sampling as a solution. Second, inter-individual variability in treatment response due to host factors highlights the need for genotype- and microbiota-based stratification for personalized protocols. Third, short follow-up periods fail to capture long-term safety and durability, advocating for robust, long-term monitoring frameworks. Integrated management combining host genetics and microbiome profiles is presented as an overarching opportunity.

## 3 Core molecular mechanisms of gut microbiota involvement in MASLD pathogenesis

### 3.1 Intestinal barrier dysfunction and endotoxemia

In metabolic dysfunction-associated steatotic liver disease (MASLD), the integrity of the intestinal barrier is critically compromised, primarily through the downregulation of tight junction proteins such as zonula occludens-1 (ZO-1) and occludin. This disruption increases intestinal mucosal permeability, facilitating the translocation of gut bacteria and their structural components, notably lipopolysaccharide (LPS), into the portal circulation and subsequently to the liver. Microbial components may also reach host tissues in a vesicle-associated form. Bacteria-derived extracellular vesicles can encapsulate LPS, proteins, lipids, and nucleic acids, potentially protecting these cargoes from degradation and facilitating their interaction with intestinal and hepatic cells (Villard et al., 2021; Qin et al., 2025). Thus, microbial translocation across a compromised intestinal barrier may involve not only whole bacteria and freely circulating molecular products but also biologically active vesicular carriers. The elevated LPS levels, a hallmark of endotoxemia, activate hepatic Kupffer cells via the Toll-like receptor 4 (TLR4) signaling pathway. This activation triggers a pro-inflammatory cascade characterized by the release of cytokines including tumor necrosis factor-alpha (TNF-α) and interleukin-6 (IL-6), which drive hepatic inflammation and fibrogenesis. The gut microbiota dysbiosis observed in MASLD exacerbates this process by promoting the overgrowth of pathogenic bacteria and reducing beneficial commensals, thereby perpetuating intestinal barrier damage. This creates a vicious cycle of dysbiosis, increased gut permeability (“leaky gut”), and hepatic inflammation, reinforcing the progression of MASLD. Accordingly, restoration of intestinal barrier integrity and reduction of microbial translocation represent mechanistically plausible intervention points, although the extent to which these approaches improve validated liver-centered outcomes in patients with MASLD remains to be established (Lau et al., 2025; Koning et al., 2023; Yu et al., 2021; Luo et al., 2023).

### 3.2 Bile acid dysregulation and compartment-specific FXR–TGR5 signaling

The gut microbiota is a major determinant of bile acid (BA) composition and signaling along the gut–liver axis. Primary BAs, principally cholic acid (CA) and chenodeoxycholic acid (CDCA) in humans, are synthesized in hepatocytes and subsequently conjugated with glycine or taurine. After secretion into the intestine, microbial bile salt hydrolases (BSHs) mediate deconjugation, while 7α-dehydroxylation and epimerization generate secondary BAs such as deoxycholic acid (DCA), lithocholic acid (LCA), and ursodeoxycholic acid (UDCA) (Sun et al., 2021; Simbrunner et al., 2021; Fleishman and Kumar, 2024). These transformations are functionally important because individual BA species differ substantially in their ability to activate the nuclear receptor farnesoid X receptor (FXR) and the membrane G protein-coupled bile acid receptor TGR5 (GPBAR1), meaning that microbiota-induced changes in BA composition can alter receptor signaling even in the absence of major changes in the total BA pool (Simbrunner et al., 2021; Fleishman and Kumar, 2024).

Importantly, intestinal and hepatic FXR signaling should be distinguished because they regulate BA homeostasis through related but anatomically distinct mechanisms. In ileal enterocytes, BA-mediated FXR activation induces fibroblast growth factor 19 (FGF19; FGF15 in mice), which is released into the portal circulation and binds the FGFR4/β-Klotho receptor complex on hepatocytes. This enterohepatic endocrine pathway strongly suppresses CYP7A1-mediated BA synthesis and also contributes to the regulation of glucose and lipid metabolism (Sun et al., 2021; Simbrunner et al., 2021). By contrast, hepatic FXR directly induces the small heterodimer partner (SHP), which represses transcriptional regulators of CYP7A1 and CYP8B1, thereby limiting *de novo* BA synthesis. Hepatic FXR also promotes biliary BA export through bile salt export pump (BSEP) regulation and restricts hepatic BA uptake, protecting hepatocytes against excessive intracellular BA accumulation (Sun et al., 2021; Simbrunner et al., 2021; Fleishman and Kumar, 2024). Thus, the intestinal FXR–FGF19 axis and hepatic FXR–SHP pathway constitute complementary but non-equivalent feedback systems, and referring simply to “FXR activation” may obscure important tissue-specific effects.

BA structure further determines the direction and magnitude of FXR signaling. Among the major unconjugated human BAs, CDCA is the most potent endogenous FXR agonist, followed approximately by DCA, LCA, and CA, whereas UDCA has very low FXR efficacy and may behave functionally as a weak partial agonist or antagonist in the presence of stronger ligands (Fleishman and Kumar, 2024). Therefore, the distinction between primary and secondary BAs cannot be reduced to a simple agonist-versus-antagonist classification: the primary BA CDCA is a strong FXR agonist, whereas the primary BA CA is substantially weaker, and the secondary BAs DCA and LCA retain FXR agonistic activity but differ in potency (Fleishman and Kumar, 2024). Species differences are also important. In mice, tauro-α-muricholic acid and tauro-β-muricholic acid are endogenous intestinal FXR antagonists whose concentrations are regulated by gut microbial BSH activity (Sayin et al., 2013). Because muricholic acids constitute a much larger proportion of the murine than the human BA pool, mechanisms based on intestinal FXR antagonism in mouse models should not be directly extrapolated to human MASLD (Fleishman and Kumar, 2024; Sayin et al., 2013).

TGR5 provides a second major pathway through which microbiota-dependent BA remodeling influences host metabolism. In contrast to FXR, TGR5 is a cell-surface G protein-coupled receptor that signals primarily through cAMP and is expressed in intestinal enteroendocrine L cells, immune cells including macrophages, and biliary epithelial cells (Simbrunner et al., 2021; Fleishman and Kumar, 2024). The relative agonist potency of endogenous BAs at TGR5 differs from that at FXR, with LCA and DCA being particularly potent TGR5 agonists, followed by CDCA and CA (Simbrunner et al., 2021; Fleishman and Kumar, 2024). Consequently, microbial 7α-dehydroxylation of primary BAs to secondary BAs can shift signaling away from predominantly FXR-directed effects toward stronger TGR5 activation. In intestinal L cells, TGR5 activation stimulates glucagon-like peptide-1 (GLP-1) secretion and thereby contributes to improved glucose handling, whereas activation in macrophages can suppress pro-inflammatory signaling (Simbrunner et al., 2021; Fleishman and Kumar, 2024). These effects indicate that BA-mediated regulation of MASLD reflects the integrated balance of FXR and TGR5 signaling rather than FXR activity alone.

This receptor-level distinction is particularly relevant when interpreting microbiota-targeted interventions. Alterations in microbial BSH and 7α-dehydroxylase activities can simultaneously change BA deconjugation, secondary BA formation, and the relative activation of intestinal FXR, hepatic FXR, and TGR5. Experimental interventions such as vertical sleeve gastrectomy (VSG), dietary anthocyanins, and microbiota-modulating natural compounds have been reported to remodel BA profiles and modify FXR-dependent signaling, with associated improvements in hepatic steatosis and metabolic homeostasis (Chen et al., 2026; Zhai et al., 2022; Hu et al., 2026; Liu et al., 2023). However, these effects should not be interpreted as evidence that generalized FXR activation is uniformly beneficial. Experimental studies indicate that intestinal FXR inhibition can also improve metabolic phenotypes under specific conditions, partly through suppression of intestinal ceramide production, whereas hepatic or systemic FXR activation may exert different metabolic effects (Fleishman and Kumar, 2024; Sayin et al., 2013). Therefore, future MASLD studies should quantify individual BA species and evaluate intestinal FXR–FGF19, hepatic FXR–SHP, and TGR5 signaling separately rather than using total BA concentrations or global “FXR activation” as surrogate measures. Such compartment- and ligand-specific analyses will be essential for determining how microbiota-driven BA remodeling contributes to MASLD and for designing receptor-selective therapeutic strategies (Sun et al., 2021; Simbrunner et al., 2021; Fleishman and Kumar, 2024).

### 3.3 Short-chain fatty acid metabolism abnormalities and energy homeostasis regulation

Short-chain fatty acids (SCFAs), including acetate, propionate, and butyrate, are key metabolites produced by gut microbial fermentation of dietary fibers. SCFAs modulate host energy metabolism and inflammatory responses through activation of G protein-coupled receptors such as GPR41 and GPR43 and inhibition of histone deacetylases. In MASLD, the abundance of butyrate-producing bacteria, such as Faecalibacterium and Roseburia, is often diminished, leading to decreased butyrate levels in the gut and liver. This reduction compromises the maintenance of intestinal barrier integrity and anti-inflammatory effects, exacerbating hepatic steatosis and inflammation. Furthermore, microbial modulation of SCFA levels influences hepatic lipid metabolism directly; for instance, increased propionate levels, facilitated by gut microbiota remodeling, activate the GPR43-Ca^2+^-CAMKII-ATGL signaling pathway, promoting hepatic lipolysis and mitochondrial β-oxidation. Interventions that restore SCFA-producing bacteria or supplement SCFAs have demonstrated efficacy in improving metabolic parameters and reducing liver fat accumulation in MASLD models. Thus, SCFA metabolism represents a crucial link between gut microbiota and host energy homeostasis, with its dysregulation contributing to MASLD development (Du et al., 2025; Yang, X. et al., 2026; Zhong et al., 2024).

### 3.4 Tryptophan metabolism and aryl hydrocarbon receptor pathway

Gut microbiota-mediated metabolism of dietary tryptophan generates indole derivatives that serve as ligands for the aryl hydrocarbon receptor (AHR), a transcription factor integral to intestinal immune homeostasis and hepatic inflammatory regulation. In MASLD, alterations in tryptophan metabolism and AHR signaling disrupt the gut-liver immune axis, contributing to disease progression. For example, ginsenoside Rg1 has been shown to modulate gut microbiota composition, enhancing the production of 5-hydroxyindoleacetic acid (5-HIAA), an AHR agonist, thereby activating AHR signaling. This activation strengthens IL-22-mediated intestinal immune barrier function, reducing hepatic inflammation and lipid deposition. Dysregulation of the tryptophan-AHR pathway constitutes a critical component of the “microbiota-gut-immune-liver” axis in MASLD, and targeting this pathway offers promising anti-inflammatory and hepatoprotective therapeutic potential. Maternal supplementation with indole derivatives has also produced long-term protective effects in experimental offspring models through AHR-dependent modulation of hepatic lipid metabolism; however, these findings remain preclinical and do not yet establish therapeutic efficacy in human MASLD (Du et al., 2025; Mandala et al., 2026; Yi et al., 2026).

### 3.5 Oxidative stress, lipid peroxidation, and mitochondrial dysfunction

The gut microbiota influences hepatic oxidative stress through multiple mechanisms, including promoting bacterial translocation and chronic inflammation, altering bile acid composition that affects lipid metabolic signaling, regulating hepatocyte ferroptosis, and modulating metabolites such as trimethylamine N-oxide (TMAO) that induce reactive oxygen species (ROS) production and platelet activation. Microbiota-derived metabolites like TMAO exacerbate oxidative damage and contribute to the progression of metabolic dysfunction-associated steatohepatitis (MASH). Additionally, gut microbiota impacts mitochondrial function in hepatocytes, a critical target in MASLD pathophysiology. Mitochondrial dysfunction manifests as impaired fatty acid β-oxidation, excessive ROS generation, and ATP depletion, all of which are closely linked to hepatocellular injury. Environmental factors such as air pollution, smoking, alcohol consumption, and sedentary lifestyle further modulate gut microbiota composition, indirectly aggravating oxidative stress and tissue damage in MASLD. Therapeutic strategies aimed at restoring mitochondrial function and reducing oxidative stress through modulation of the gut microbiota and its metabolites are emerging as promising avenues for MASLD management (Yu et al., 2021).

### 3.6 Gut microbiota-metabolite-target regulatory network

Integrative analyses employing Mendelian randomization and multi-omics approaches have begun to elucidate the complex regulatory network linking gut microbiota, microbial metabolites, and host molecular targets in MASLD. Eleven microbial taxa have been identified with causal associations to MASLD, alongside 19 microbial-derived metabolites implicated in disease modulation. Network analyses reveal that these metabolites predominantly target pathways involved in cholesterol metabolism, peroxisome proliferator-activated receptor (PPAR) signaling, and hypoxia-inducible factor 1 (HIF-1) signaling. Key genes such as angiotensin-converting enzyme (ACE), 3-hydroxy-3-methylglutaryl-CoA reductase (HMGCR), PPAR alpha (PPARA), and PPAR gamma (PPARG) have been validated in MASLD animal models as critical nodes within this network. The resulting network supports a systems-level model in which microbial metabolites converge on host pathways involved in lipid metabolism, inflammation, and cellular stress. Network-derived targets should nevertheless be regarded as hypotheses for functional validation rather than immediate therapeutic candidates, particularly when their inferred relationships are based primarily on computational or genetic association analyses (Figure 2) (Zhang, D. Y. et al., 2025; Li, Z. et al., 2026).

**Figure 2:**
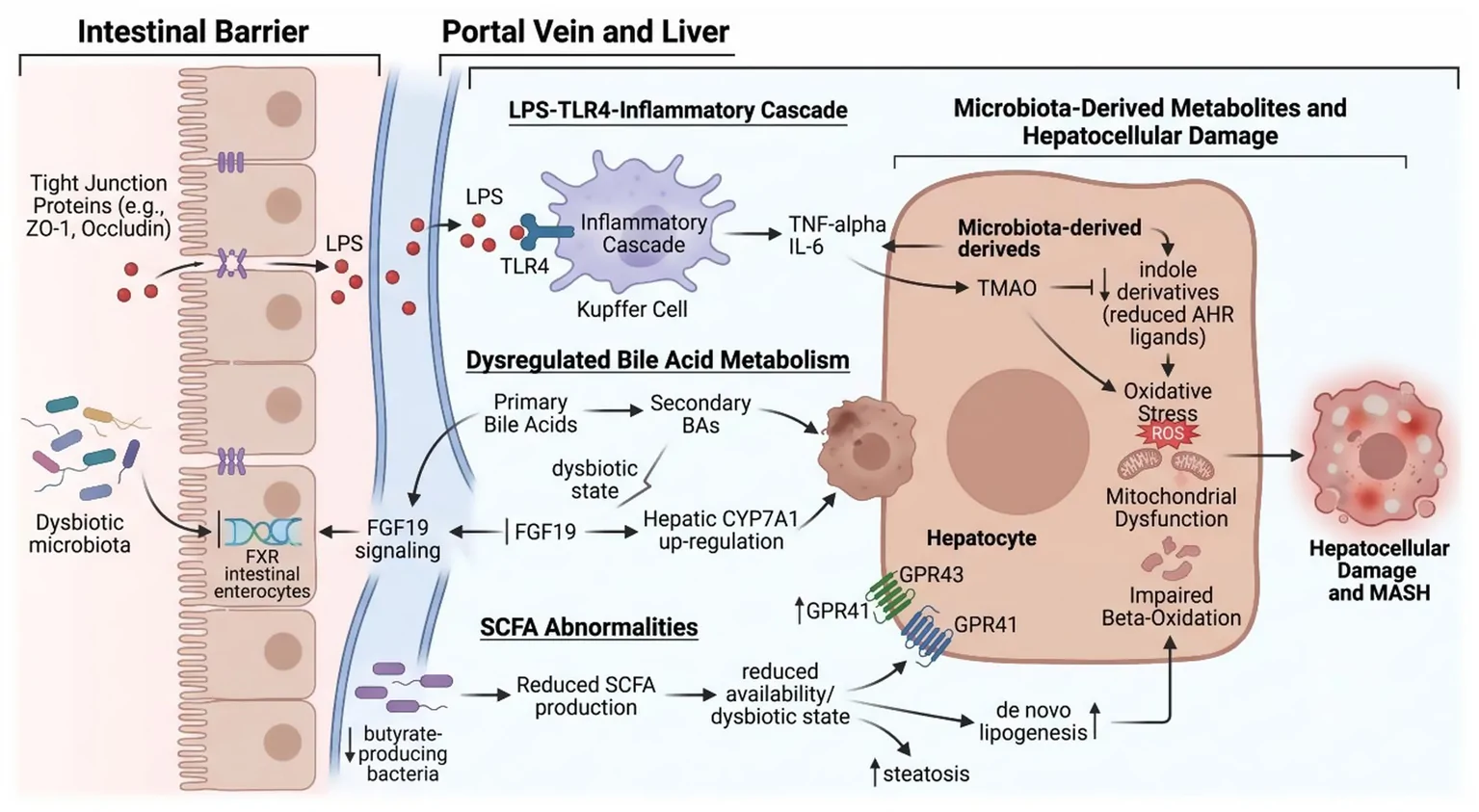
Multi-omics integration for precision medicine in MASLD. The flowchart details a comprehensive analytical workflow for studying metabolic dysfunction-associated steatotic liver disease (MASLD). It begins with sample collection (fecal, serum, liver tissue). Data is generated through three analytical layers: metagenomics (16S rRNA gene, WMS), metabolomics (LC-MS/MS for bile acids, SCFAs, TMAO), and host genetics/epigenetics (MR, PNPLA3). These layers are integrated using association networks and Mendelian randomization to infer causality. Finally, the findings are validated through functional studies in model systems (FMT, antibiotics) and translated into precision microbiome interventions.

## 4 External regulatory factors affecting gut microbiota in MASLD

### 4.1 Dietary patterns and nutrient composition

Diet is the primary environmental determinant shaping the composition and function of the gut microbiota, profoundly influencing the pathogenesis and progression of metabolic dysfunction-associated steatotic liver disease (MASLD). Western dietary patterns characterized by high fat and high sugar intake promote the establishment of a pro-inflammatory microbial ecosystem that exacerbates metabolic disturbances and hepatic steatosis. Such diets induce dysbiosis marked by an increased abundance of endotoxin-producing bacteria and reduced microbial diversity, which compromises intestinal barrier integrity and facilitates systemic endotoxemia, thereby aggravating liver inflammation and fat accumulation (Lau et al., 2025; Benedé-Ubieto et al., 2024). Conversely, diets rich in dietary fiber, plant polyphenols, and fermented foods foster the growth of beneficial bacteria, including short-chain fatty acid (SCFA) producers such as Lachnospiraceae and Ruminococcaceae, which exert anti-inflammatory effects and enhance gut barrier function (Guo et al., 2026; Ni et al., 2023). Fermented foods, abundant in probiotics, prebiotics, SCFAs, and bioactive compounds, contribute to microbial diversity enhancement, strengthen intestinal tight junctions, and modulate bile acid and lipid metabolism, collectively mitigating hepatic steatosis and inflammation (Lau et al., 2025; Ma et al., 2025). Furthermore, dietary intake of nicotinamide adenine dinucleotide (NAD) precursors interacts synergistically with the gut microbiota to remodel specific bacterial taxa. Notably, the genus Prevotella shows abundance changes closely linked to NAD metabolism and branched-chain amino acid-related fatty acid metabolic pathways, suggesting a mechanistic link between nutrient availability, microbial metabolism, and host mitochondrial function in MASLD (Feng et al., 2024). Dietary effects on MASLD are therefore likely mediated in part through changes in microbial substrates and metabolic outputs, although the magnitude and direction of these effects depend on dietary composition, baseline microbiota, and host metabolic status.

### 4.2 Host genetic factors and gene-microbiota interactions

Host genetic polymorphisms significantly influence gut microbiota composition and function through modulation of immune responses, bile acid metabolism, and intestinal mucus secretion, thereby indirectly affecting MASLD development. A prominent example is the PNPLA3 rs738409 variant, a well-established genetic risk factor for MASLD, which is associated with distinct patterns of gut microbial dysbiosis. Carriers of the PNPLA3 rs738409 CC/CG genotypes exhibit a marked reduction in beneficial bacterial genera such as Blautia and members of the Ruminococcaceae family, taxa known for their SCFA production and anti-inflammatory properties (Jiang et al., 2026). These microbial alterations correlate negatively with clinical parameters including controlled attenuation parameter (CAP), body mass index (BMI), blood glucose, and triglyceride levels, suggesting that the genetic predisposition modulates metabolic phenotypes via microbiota-mediated mechanisms (Stols-Gonçalves et al., 2023). The interplay between host genetics and microbiota composition implies that personalized microbiota-targeted interventions, tailored to the host’s genetic background, may enhance therapeutic efficacy. For instance, MASLD patients harboring the CC/CG genotypes of PNPLA3 may derive greater benefit from dietary modifications and probiotic supplementation aimed at restoring Blautia and Ruminococcaceae abundance, thereby improving metabolic and hepatic outcomes (Jiang et al., 2026; Stols-Gonçalves et al., 2023). These observations provide a rationale for incorporating host genotype into studies of microbiome heterogeneity and treatment response, although genotype-specific microbiota interventions require prospective clinical validation before they can be incorporated into therapeutic decision-making.

### 4.3 Sex-related differences in gut microbiota and MASLD susceptibility

Biological sex is an increasingly recognized modifier of both MASLD susceptibility and gut microbial ecology. Epidemiologically, MASLD is generally more prevalent in men than in premenopausal women, whereas disease susceptibility increases substantially in women after menopause, implicating sex hormones, particularly estrogen signaling, in the regulation of hepatic lipid metabolism, inflammatory responses, and fibrosis (Booijink et al., 2024). Because sex hormones also influence intestinal microbial composition, part of the sex disparity in MASLD may involve bidirectional interactions among the gut microbiota, host metabolism, and endocrine signaling.

Human microbiome studies provide evidence that MASLD-associated microbial signatures may differ between men and women. In a large study of urban Chinese adults conducted under the former NAFLD diagnostic framework, male cases exhibited lower microbial α-diversity than male controls, whereas female cases showed higher α-diversity than female controls; several taxa, including Dialister and Bifidobacterium species, also displayed opposite or sex-dependent associations with disease status (Shi et al., 2021). Significant disease-by-sex interactions were observed for both microbial diversity and individual taxa (Shi et al., 2021). More recently, analysis of 98 patients with MASLD and 45 controls from the Fatty Liver in Obesity study identified sex-dependent differences in microbial diversity and functional signatures. Christensenella and Limosilactobacillus were among the taxa most strongly associated with MASLD in women, whereas Beduinibacterium and Anaerotruncus contributed more prominently to the male microbial signature (Mogna-Peláez et al., 2024). Sex-specific microbiome-based classification also showed different predictive performance between women and men, further suggesting that a single microbial signature may not be equally informative across sexes (Mogna-Peláez et al., 2024).

Experimental evidence provides additional mechanistic support but should be distinguished from human observations. In a rat model of MASLD, microbial composition differed markedly between males and females in both the jejunum and colon, with greater β-diversity in female animals and higher hepatic lipopolysaccharide levels and TLR4 expression in males (Pirola et al., 2025). These findings suggest a potential sex-dependent difference in microbiota-derived inflammatory signaling, although their relevance to human MASLD requires validation. Sex-related microbiome variation may reflect differences in estrogen and androgen signaling, adipose tissue distribution, bile acid metabolism, immune tone, diet, and other behavioral or environmental exposures (Booijink et al., 2024; Pirola et al., 2025).

Importantly, sex-specific microbiome findings remain heterogeneous, and many existing MASLD cohorts were not designed or sufficiently powered to test sex-by-microbiome interactions. Menopausal status represents an additional source of heterogeneity because chronological age alone does not adequately capture the endocrine transition associated with estrogen loss. Future microbiome studies in MASLD should therefore prespecify sex-stratified analyses and record menopausal status, menopausal hormone therapy, age, metabolic phenotype, and relevant medications. Sex and menopausal status should also be evaluated as potential effect modifiers in microbiota-targeted intervention trials rather than treated solely as adjustment covariates (Booijink et al., 2024; Shi et al., 2021; Mogna-Peláez et al., 2024). Such approaches may help determine whether sex-specific microbial signatures contribute causally to differential MASLD susceptibility or primarily reflect underlying hormonal and metabolic differences.

### 4.4 Lifestyle and environmental exposure factors

Lifestyle behaviors and environmental exposures critically modulate gut microbiota diversity and functionality, influencing MASLD progression. Physical inactivity is linked to decreased microbial diversity and a reduction in butyrate-producing bacteria, which are essential for maintaining intestinal barrier integrity and anti-inflammatory homeostasis. Regular exercise independently improves gut microbial composition by increasing SCFA production, thereby alleviating metabolic dysregulation associated with MASLD, regardless of dietary intake (Ni et al., 2023; Ahmed et al., 2021). Conversely, smoking and excessive alcohol consumption synergistically induce gut dysbiosis, increase intestinal permeability, and promote oxidative stress, collectively exacerbating hepatic inflammation and fibrosis in MASLD (Benedé-Ubieto et al., 2026; Yan et al., 2024). Moreover, exposure to air pollutants has emerged as a novel environmental risk factor that disrupts gut microbial communities and metabolic functions, triggering inflammatory pathways and impairing intestinal immune homeostasis, thereby elevating MASLD susceptibility (Wu et al., 2025). From a clinical perspective, lifestyle and environmental exposures should therefore be considered both major covariates in microbiome studies and modifiable components of comprehensive MASLD management. Their effects on the gut microbiome should be interpreted within the broader metabolic consequences of exercise, smoking, alcohol exposure, and environmental pollutants rather than being attributed exclusively to microbiota-mediated mechanisms (Figure 3) (Ni et al., 2023; Ahmed et al., 2021; Benedé-Ubieto et al., 2026; Yan et al., 2024; Wu et al., 2025).

**Figure 3:**
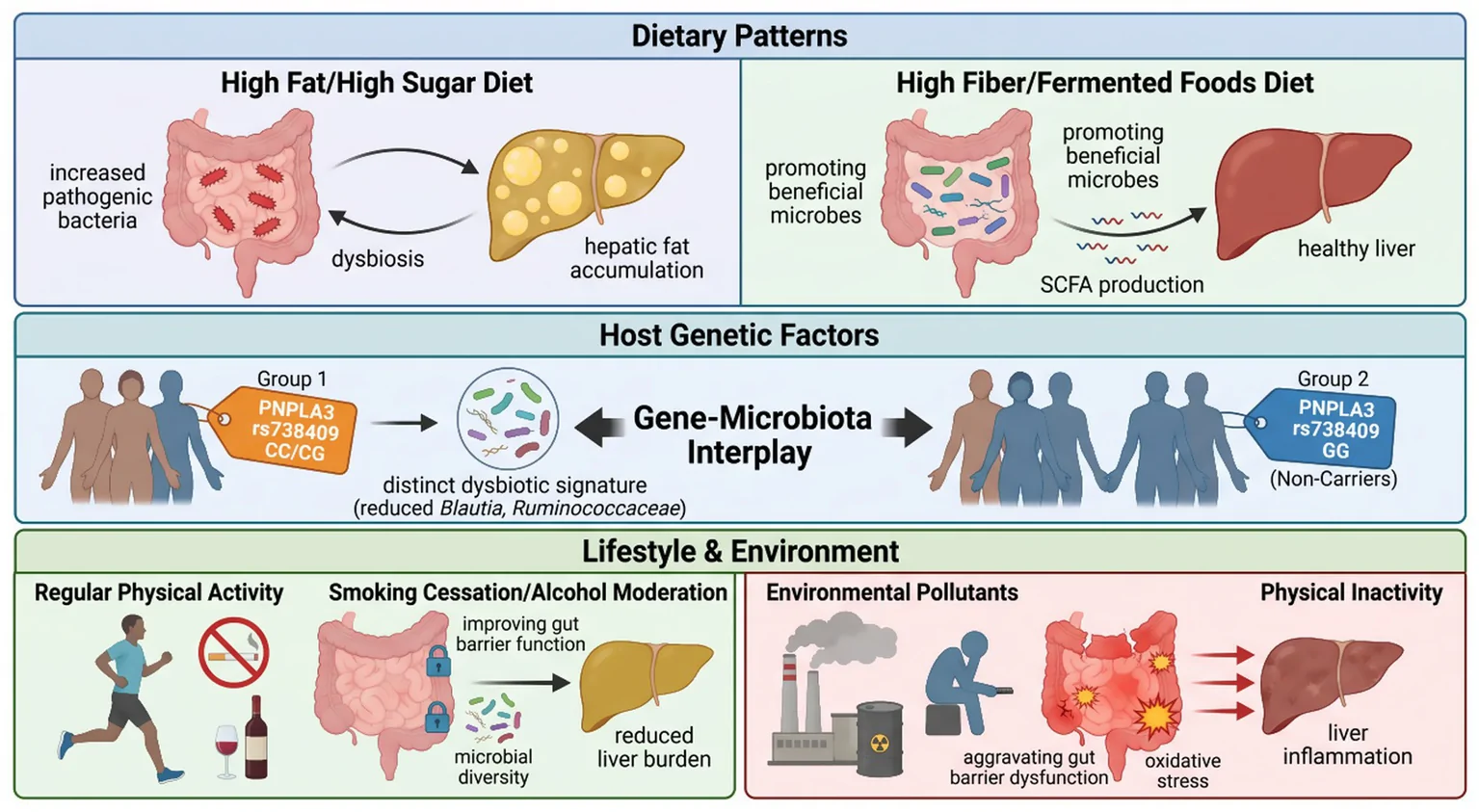
Impact of diet, host genetics, lifestyle, and environment on gut-liver health. This diagram illustrates how various factors influence the gut microbiota and, subsequently, liver health. High-fat/high-sugar diets promote pathogenic bacteria, leading to dysbiosis and hepatic fat accumulation, whereas high-fiber/fermented foods promote beneficial microbes and SCFA production, supporting a healthy liver. Host genetic factors, specifically PNPLA3 genotype, influence the gene-microbiota interplay and can create a distinct dysbiotic signature. Lifestyle choices like physical activity and alcohol moderation improve gut function and reduce liver burden, while environmental pollutants and physical inactivity aggravate gut dysfunction and cause liver inflammation.

## 5 Microbiota-targeted strategies for MASLD: from preclinical evidence to clinical translation

The level of evidence supporting microbiota-targeted interventions in MASLD varies substantially across therapeutic categories and should be interpreted accordingly. Mechanistic efficacy has been demonstrated predominantly in cell and animal models for several approaches, whereas human evidence remains limited or heterogeneous for probiotics, synbiotics, and fecal microbiota transplantation (FMT) (Groenewegen et al., 2025; Lau et al., 2025; Saeed et al., 2025). In particular, most evidence for traditional Chinese medicine (TCM)-derived bioactive compounds and several metabolite-directed interventions remains preclinical. Therefore, improvement of hepatic steatosis, inflammation, or microbial composition in experimental models should not be interpreted as evidence of clinical efficacy or readiness for routine use. Translation into clinical practice requires demonstration of reproducible efficacy in appropriately powered human trials using validated liver-centered endpoints, together with evidence of target engagement, long-term safety, and durability of response (Saeed et al., 2025; Li, M. et al., 2026).

### 5.1 Probiotics, prebiotics, and synbiotics

Probiotics, prebiotics, and synbiotics have been investigated as microbiota-directed interventions in MASLD; however, their clinical efficacy remains uncertain and appears to depend on formulation, host characteristics, and study design. Probiotics, particularly specific strains of Lactobacillus and Bifidobacterium, exert beneficial effects by competitively inhibiting pathogenic bacterial colonization, producing antimicrobial substances, enhancing intestinal barrier integrity, and modulating host immune responses. Experimental studies in MASLD animal models have demonstrated that administration of these probiotic strains reduces hepatic steatosis and inflammation, suggesting their potential to mitigate liver injury associated with MASLD. Human evidence, however, is considerably less consistent than preclinical findings. Recent quantitative syntheses have reached partially discordant conclusions. One systematic review and meta-analysis of randomized controlled trials found that probiotics alone did not significantly improve the major clinical indicators of MASLD, whereas synbiotic interventions were associated with improvements in hepatic steatosis and several biochemical and inflammatory parameters (Wu et al., 2024). In contrast, a more recent meta-analysis reported modest reductions in aminotransferases and improvements in lipid profiles following probiotic supplementation, although substantial heterogeneity remained for several outcomes (Lu et al., 2025). These discrepancies likely reflect both biological and methodological variability rather than a uniform class effect of microbiota-directed supplementation.

First, probiotic effects are highly strain- and formulation-specific, whereas clinical studies frequently differ in the bacterial species and strains used, single- versus multi-strain formulations, viable dose, treatment duration, and whether probiotics are administered alone or in combination with prebiotics (Saeed et al., 2025; Wu et al., 2024; Lu et al., 2025). Second, recipient-related factors—including age, obesity, type 2 diabetes, MASLD stage, habitual diet, concomitant medications, and baseline gut microbial composition—may determine whether an administered strain can engraft or exert the intended metabolic effect (Lau et al., 2025; Saeed et al., 2025). Third, considerable heterogeneity exists in outcome assessment: many studies primarily evaluate aminotransferases, HOMA-IR, lipid parameters, or ultrasonographic steatosis rather than quantitative liver-centered endpoints, making biologically modest effects difficult to distinguish from clinically meaningful disease modification (Wu et al., 2024; Lu et al., 2025). Moreover, relatively few trials simultaneously demonstrate microbiome or metabolome changes, so an absence of clinical benefit cannot readily be distinguished from inadequate microbial target engagement (Saeed et al., 2025).

Future trials should therefore move beyond generic standardization toward mechanism-informed patient selection and intervention design. Probiotic products should be reported at the strain level with defined viable doses, while background diet and microbiome-modifying medications should be prospectively documented or controlled. Participants should be stratified according to MASLD stage, metabolic phenotype, baseline microbial or functional signatures, biological sex, and, in women, menopausal status, because sex-related microbial differences may modify both disease susceptibility and response to microbiota-directed interventions (Booijink et al., 2024; Shi et al., 2021; Mogna-Peláez et al., 2024). Serial metagenomic and metabolomic analyses should be incorporated to verify microbial engraftment and functional target engagement, alongside standardized liver-centered outcomes such as MRI-proton density fat fraction and validated fibrosis measurements. Predefined responder analyses may further identify microbial or metabolic signatures associated with therapeutic benefit, providing a testable basis for precision probiotic or synbiotic therapy rather than an empirical “one-size-fits-all” approach (Saeed et al., 2025; Borges-Canha et al., 2025; Li, Z. et al., 2026).

### 5.2 Fecal microbiota transplantation

Fecal microbiota transplantation (FMT) has been investigated as a strategy to modify gut microbial composition and function in MASLD through the transfer of donor microbial communities; however, its clinical efficacy remains uncertain. The rationale for this approach is to modify dysbiotic microbial communities and potentially alter microbiota-dependent metabolic and immune functions. Preclinical studies have provided compelling evidence supporting FMT’s efficacy; for instance, transplantation of fecal microbiota from rats subjected to vertical sleeve gastrectomy (VSG) or duodenal-jejunal bypass (DJB) surgeries into MASLD model rats resulted in significant attenuation of hepatic lipid accumulation, improved insulin sensitivity, and reduced inflammatory markers. These transfer experiments support a microbiota-mediated component of the metabolic response to bariatric surgery; however, because they were performed in animal recipients, they establish mechanistic plausibility rather than clinical efficacy of FMT. The critical importance of an intact and viable microbial community was confirmed through antibiotic depletion experiments, where eradication of gut bacteria abolished the protective effects of FMT, highlighting that the therapeutic benefits depend on the engraftment of live microbes and their metabolic activities. In contrast to the relatively consistent preclinical evidence, clinical studies of FMT in MASLD have produced discordant results. In a randomized controlled trial by Craven et al., allogenic FMT did not significantly improve insulin resistance measured by HOMA-IR or hepatic fat quantified by MRI-proton density fat fraction, although intestinal permeability improved in the subgroup of patients with abnormal permeability at baseline (Craven et al., 2020). Conversely, Xue et al., in a study conducted under the former NAFLD diagnostic framework, reported reductions in hepatic fat accumulation after FMT and observed more pronounced reconstruction of the gut microbiota in lean than in obese participants (Xue et al., 2022). Interpretation of this study, however, is complicated by the use of probiotics as an active comparator, concomitant dietary and exercise interventions, and a relatively short follow-up period (Xue et al., 2022). More recently, a randomized trial of consecutive FMT in MASLD found no significant overall improvement in hepatic steatosis, glucose tolerance, or liver biochemistry (Groenewegen et al., 2025). Notably, participants in this trial had relatively high baseline microbial α-diversity, and donor microbiota engraftment appeared donor-specific but was not clearly associated with clinical response (Groenewegen et al., 2025).

These apparently conflicting findings suggest that FMT efficacy may depend less on the administration of a “healthy microbiota” per se than on the presence of a correctable recipient microbial phenotype and the establishment of durable, functionally relevant donor engraftment (Groenewegen et al., 2025; Saeed et al., 2025; Stols-Gonçalves et al., 2023). Differences in baseline microbial diversity, intestinal barrier dysfunction, obesity and metabolic phenotype, donor microbial characteristics, transplantation route and dosing frequency, comparator interventions, follow-up duration, and clinical endpoints are therefore plausible contributors to between-trial inconsistency (Groenewegen et al., 2025; Saeed et al., 2025; Craven et al., 2020; Xue et al., 2022). Importantly, changes in microbial composition alone should not be considered evidence of therapeutic efficacy unless they are linked to relevant functional metabolic changes and liver-centered outcomes (Groenewegen et al., 2025; Stols-Gonçalves et al., 2023).

Future FMT trials should consequently adopt biologically enriched rather than unselected MASLD cohorts. Patients with low microbial diversity, defined dysbiosis, or impaired intestinal barrier function may represent candidate responder subgroups that warrant prospective evaluation (Groenewegen et al., 2025; Craven et al., 2020). Donor material should undergo comprehensive taxonomic and functional characterization in addition to pathogen and antimicrobial-resistance screening, while donor-to-recipient strain engraftment and associated changes in bile acids, short-chain fatty acids, and other microbial metabolites should be monitored longitudinally (Groenewegen et al., 2025; Saeed et al., 2025; Stols-Gonçalves et al., 2023). Trials should also directly compare defined transplantation routes and dosing schedules and employ harmonized hepatic endpoints and sufficiently long follow-up periods. Ultimately, prospective donor–recipient matching based on reproducible microbial or functional signatures may be required to determine whether FMT can be transformed from an empiric intervention into a precision microbiome therapy (Saeed et al., 2025; Li, Z. et al., 2026). Until reproducible benefits on validated liver-centered outcomes are demonstrated, FMT should be regarded as an investigational strategy for MASLD rather than an established treatment (Groenewegen et al., 2025; Saeed et al., 2025).

### 5.3 TCM-derived bioactive compounds and natural products: predominantly preclinical evidence

Current evidence supporting TCM-derived bioactive compounds and natural products as microbiota-targeted therapies for MASLD is predominantly preclinical. Several experimental studies nevertheless provide biologically plausible mechanisms linking these agents to the gut-liver axis. In diet-induced murine MASLD, ginsenoside Rg1 was shown to reshape the gut microbiota and alter tryptophan metabolism, increasing 5-hydroxyindoleacetic acid and activating the AHR/IL-22 pathway; microbiota depletion and fecal transfer experiments further supported a microbiota-dependent component of its hepatoprotective effect (Wang, Z. et al., 2025). Similarly, Alisol B attenuated hepatic steatosis in high-fat diet-fed mice while modifying gut microbial composition and hepatic purine metabolism, including inhibition of xanthine oxidase activity (Yiyou et al., 2025). Geniposidic acid, another TCM-derived compound, improved steatosis, oxidative stress, inflammation, and fibrosis in experimental MASLD through activation of SIRT6 and downstream PPARα signaling, although this evidence was also derived from cellular and mouse models (Zhou et al., 2025).

Complex herbal formulations have likewise shown microbiome-related effects in experimental systems. Wuling Powder reduced hepatic lipid accumulation and inflammatory injury in a high-fat diet-induced rat model and was associated with alterations in gut microbial composition and multiple metabolic pathways (Yang, H. et al., 2026). ACT001 has also been evaluated in two murine MASLD models, in which it improved hepatic lipid accumulation and intestinal barrier integrity while modulating gut microbiota, bile acid composition, and the intestinal FXR-FGF15 axis (Niu et al., 2025). Collectively, these studies provide mechanistic evidence that TCM-derived compounds can influence interconnected microbial, metabolic, and inflammatory pathways; however, they should be regarded as proof-of-concept rather than evidence of clinical efficacy.

The translational gap remains substantial. For the specific agents discussed above, the available microbiome-centered evidence is derived largely from animal and cellular experiments, and adequately powered human MASLD trials demonstrating microbiota-mediated improvements in validated liver outcomes remain lacking. Several factors further limit direct extrapolation from experimental models to patients, including interspecies differences in gut microbial ecology and bile acid metabolism, uncertainty regarding human-equivalent dosing and bioavailability, variability in the chemical composition and quality control of multi-component herbal preparations, potential herb-drug interactions, and the absence of long-term safety data. Moreover, changes in microbial composition alone cannot establish therapeutic efficacy unless they are accompanied by reproducible functional changes and clinically meaningful liver outcomes (Saeed et al., 2025; Li, M. et al., 2026).

These limitations are consistent with current clinical guidance. The EASL-EASD-EASO Clinical Practice Guidelines state that nutraceuticals, including herbal products and gut microbiota-modifying agents, cannot currently be recommended for adults with MASLD because evidence is insufficient regarding their effects on histologically or non-invasively assessed liver damage, fibrosis, liver-related outcomes, and safety (European Association for the Study of the Liver (EASL) et al., 2024). Therefore, clinical translation of TCM-derived microbiome interventions should follow a staged framework: first, rigorous chemical standardization, pharmacokinetic characterization, and toxicological assessment; second, early-phase human proof-of-mechanism studies incorporating metagenomic and metabolomic measures of target engagement; and finally, adequately powered multicenter randomized controlled trials using standardized endpoints such as MRI-proton density fat fraction, validated fibrosis measures, or histological outcomes, together with long-term safety monitoring. Until such evidence becomes available, these agents should be considered experimental therapeutic candidates rather than established microbiota-directed treatments for MASLD (Saeed et al., 2025; Li, M. et al., 2026).

### 5.4 Gut microbiota-mediated mechanisms of weight loss and metabolic surgery

Metabolic/bariatric surgery is an established treatment for eligible patients with obesity and can improve MASLD-related hepatic and metabolic outcomes (Liu et al., 2025). However, evidence that gut microbiota remodeling or bile-acid signaling causally mediates these clinical benefits is derived predominantly from experimental models (Liu et al., 2025; Saeed et al., 2025). In animal studies, vertical sleeve gastrectomy (VSG) and duodenal-jejunal bypass (DJB) alter microbial bile salt hydrolase activity, bile-acid composition, and FXR signaling. Antibiotic-mediated microbiota depletion, fecal microbiota transfer, and FXR manipulation further support a microbiota-dependent component of the postsurgical metabolic phenotype. These mechanistic experiments provide important proof-of-concept but should not be interpreted as demonstrating that microbiota remodeling is necessary or sufficient for the clinical liver benefits of bariatric surgery in humans. Future translational studies should therefore distinguish the established clinical efficacy of metabolic surgery from the still-investigational microbiome-mediated mechanisms (Liu et al., 2025; Saeed et al., 2025; Li, M. et al., 2026).

### 5.5 Novel interventions targeting microbial metabolites

Therapeutic strategies targeting microbiota-derived metabolites span a wide range of evidence levels. Among bile acid-directed approaches, pharmacological modulation of FXR signaling has progressed into human clinical development, although efficacy and safety depend on receptor selectivity, tissue distribution, and the specific disease context (Sun et al., 2021; European Association for the Study of the Liver (EASL) et al., 2024). In contrast, many strategies aimed at directly manipulating microbial metabolites remain at the preclinical or early translational stage.

For example, short-chain fatty acids (SCFAs), particularly propionate and butyrate, regulate hepatic lipid metabolism, intestinal barrier function, and immune responses through G protein-coupled receptors and other signaling pathways. Experimental studies have shown that increasing SCFA availability or restoring SCFA-producing bacteria can improve steatosis and metabolic dysfunction in animal models (Lau et al., 2025; Lee et al., 2026). However, evidence that direct SCFA supplementation produces clinically meaningful liver benefits in patients with MASLD remains limited, and differences in dose, route of administration, colonic delivery, and endogenous microbial production complicate translation.

Similarly, interventions targeting nicotinamide adenine dinucleotide (NAD) metabolism or selectively suppressing metabolically unfavorable bacterial taxa remain primarily mechanistic concepts. Experimental studies suggest that NAD precursors can improve mitochondrial function and alter gut microbial composition, whereas disease-associated bacteria may influence hepatic injury through bile acid transformation and other metabolic pathways (González-González et al., 2026; Luo et al., 2025). Nevertheless, modification of a microbial metabolite or taxon in experimental models does not establish therapeutic efficacy in humans. In particular, associations between specific organisms, such as *Ruminococcus torques*, and secondary bile acids should be regarded as candidate mechanistic targets rather than validated clinical treatment targets unless supported by prospective intervention studies.

Therefore, microbiota-derived metabolites are best viewed as a spectrum of therapeutic opportunities ranging from clinically investigated receptor-directed pharmacology to predominantly preclinical microbial or metabolite manipulation. Future studies should clearly distinguish proof-of-mechanism endpoints from clinical efficacy endpoints and should incorporate quantitative metabolomics, confirmation of biological target engagement, and validated liver-centered outcomes before these approaches are advanced toward routine MASLD treatment (Lau et al., 2025; Saeed et al., 2025; European Association for the Study of the Liver (EASL) et al., 2024; Luo et al., 2025).

### 5.6 Pharmacological therapies at the microbiome–bile acid interface: resmetirom and incretin-based drugs

Although resmetirom and incretin-based drugs are not primarily designed as microbiota-targeted therapies, emerging evidence indicates that their metabolic effects intersect with the gut microbiome and bile acid (BA) signaling. This relationship may be bidirectional: pharmacological treatment can alter nutrient delivery, intestinal physiology, microbial ecology, and BA composition, whereas the baseline microbiome and microbially modified metabolites may influence host signaling and potentially contribute to interindividual variability in therapeutic response. However, the strength of evidence differs substantially among drug classes, and direct microbiome-mediated effects in patients with MASLD/MASH remain incompletely established.

Resmetirom, a liver-directed thyroid hormone receptor-β (THR-β) agonist, primarily improves MASH through hepatic lipid oxidation, mitochondrial metabolism, and cholesterol handling, but experimental evidence also indicates effects on the enterohepatic BA pool. In two murine steatohepatitis models, resmetirom reduced the hepatic and intestinal ratio of 12α-hydroxylated to non-12α-hydroxylated BAs through suppression of CYP8B1, thereby decreasing intestinal lipid absorption; supplementation with cholic acid reversed this effect, supporting a functional contribution of BA remodeling to the metabolic action of THR-β activation (Sun et al., 2024). Evidence that the microbiome can reciprocally modify responses to this drug class is more preliminary. A related THR-β agonist, HSK31679, studied alongside MGL-3196 (resmetirom) in germ-free and specific-pathogen-free mice, showed microbiota-dependent differences in therapeutic efficacy. Cross-omics analysis further identified *Bacteroides thetaiotaomicron* and microbial glucosylceramide synthase activity as potential modifiers of THR-β agonist response (Zhang, Y. H. et al., 2025). Importantly, the human component of that study evaluated HSK31679 rather than resmetirom itself; therefore, these findings support a possible class-level pharmacomicrobiomic interaction but should not be interpreted as direct evidence that the clinical efficacy of resmetirom is microbiome-dependent (Zhang, Y. H. et al., 2025).

For incretin-based therapies, evidence for a bidirectional host–microbiome interaction is broader but remains heterogeneous. Semaglutide can alter gastrointestinal motility, food intake, nutrient flux, and metabolic status, all of which may secondarily reshape the intestinal ecosystem. In a small 12-week study of 15 patients with type 2 diabetes receiving concomitant metformin, semaglutide was associated with changes in microbial community structure, including increased Bifidobacterium and decreased Klebsiella, together with alterations in circulating metabolic profiles (Chen et al., 2025). A separate longitudinal study with serial fecal sampling found treatment-associated changes in microbial community composition without significant changes in overall microbial diversity; notably, pretreatment microbiome features predicted the subsequent HbA1c response to semaglutide, providing human evidence that the microbiome may function not only as a downstream target but also as a potential determinant of drug response (Klemets et al., 2026). Nevertheless, these cohorts were small, were not restricted to MASLD, and were potentially confounded by background medications and treatment-associated weight loss.

Preclinical experiments provide stronger evidence for microbiome-mediated effects of semaglutide. In high-fat diet-fed mice, semaglutide remodeled gut microbial composition and metabolic profiles, and fecal microbiota transfer from semaglutide-treated donors partially transferred metabolic improvements to microbiota-depleted recipients (Sun et al., 2025). BA signaling may represent an additional link between incretin therapy and the microbiome because microbial conversion of primary to secondary BAs influences endogenous GLP-1 secretion through TGR5 and FXR-dependent pathways (Simbrunner et al., 2021; Fleishman and Kumar, 2024). Conversely, pharmacologically induced changes in food intake, gastrointestinal transit, and enterohepatic BA circulation may alter the substrates available for microbial BA transformation. A recent prospective study of women with obesity, accompanied by mechanistic animal experiments, further associated weight regain after semaglutide withdrawal with reversal of microbiota-related metabolic changes; in the animal component, withdrawal was accompanied by reduced ursodeoxycholic acid levels and decreased TGR5 signaling (Wang, Z. et al., 2026). These observations support a microbiome–BA–incretin feedback model, although direct demonstration that BA remodeling mediates semaglutide efficacy in human MASLD is still lacking.

Tirzepatide, a dual glucose-dependent insulinotropic polypeptide (GIP)/GLP-1 receptor agonist, may engage a similar microbiome–BA axis. In diabetic mice with hepatic steatosis, tirzepatide increased the abundance of Akkermansia and altered the BA pool, increasing the relative abundance of BAs characterized in that experimental system as intestinal FXR antagonists relative to agonists, with concomitant suppression of intestinal FXR expression (Hu et al., 2025). These changes were associated with reduced hepatic lipid accumulation and improved insulin resistance (Hu et al., 2025). However, this evidence is preclinical, and some of the implicated BA species, particularly β-muricholic acid, are quantitatively important in mice but not humans. Accordingly, the microbiota–BA–FXR mechanism observed in these models should not yet be assumed to mediate the clinical hepatic benefits of tirzepatide.

Taken together, these findings suggest a bidirectional pharmacomicrobiomic framework in which resmetirom and incretin-based therapies may modify the gut environment and BA pool, while microbial composition and microbial metabolites may in turn influence metabolic signaling or treatment responsiveness (Zhang, Y. H. et al., 2025; Klemets et al., 2026; Simbrunner et al., 2021; Fleishman and Kumar, 2024). Nevertheless, direct drug–microbiome interactions must be distinguished from secondary microbial changes caused by weight loss, reduced caloric intake, delayed gastric emptying, altered dietary preferences, or improved glycemic control. Future MASLD trials should therefore incorporate longitudinal metagenomic and BA metabolomic profiling before and during pharmacotherapy, control for diet and microbiome-modifying medications, and test whether baseline microbial or BA signatures predict histological or imaging-based treatment responses. Mediation analyses and, where feasible, functional validation will be required to determine whether microbiome and BA changes are causal components of drug efficacy or merely biomarkers of the accompanying metabolic improvement (Figure 4).

**Figure 4:**
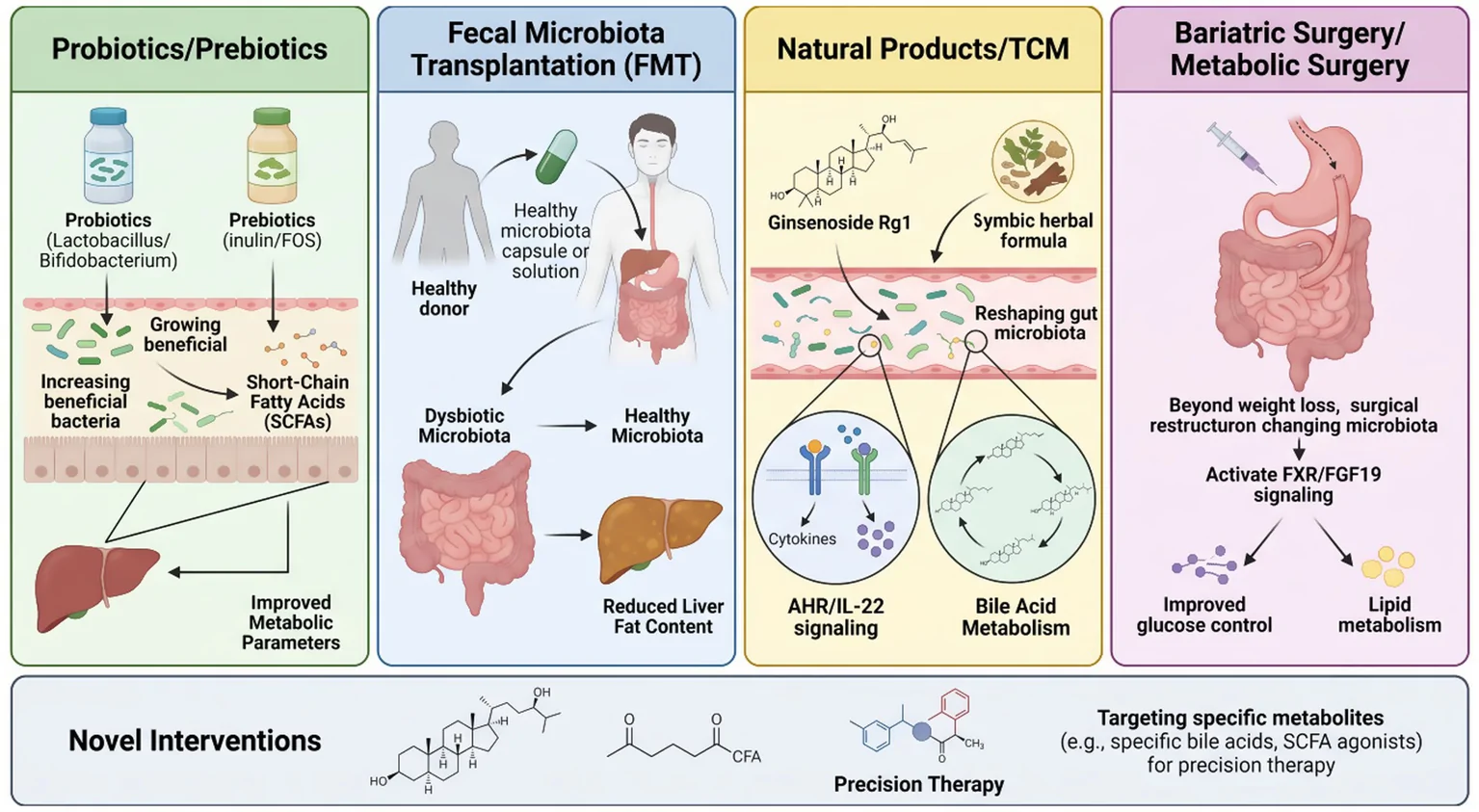
Mechanisms of gut-derived inflammation and metabolic dysregulation in liver disease. The schematic depicts the pathways by which a compromised intestinal barrier leads to hepatocellular damage and MASH. Dysbiotic microbiota produce lipopolysaccharides (LPS), which cross the barrier, bind to TLR4 on Kupffer cells, and trigger an inflammatory cascade (TNF-alpha, IL-6). Simultaneously, dysregulated bile acid metabolism reduces FXR signaling, promoting hepatic CYP7A1 up-regulation and a dysbiotic state. Additionally, reduced SCFA production contributes to steatosis through increased de novo lipogenesis and impaired beta-oxidation. These combined insults—inflammation, toxic metabolites, and altered lipid metabolism—culminate in hepatocyte stress, mitochondrial dysfunction, and hepatocellular damage.

## 6 Application of multi-omics technologies and precision microbiome analysis in MASLD research

### 6.1 Integrated strategies of metagenomics and metabolomics

The integration of metagenomics and metabolomics represents a powerful approach to comprehensively characterize the gut microbiome and its functional impact in metabolic dysfunction-associated steatotic liver disease (MASLD). 16S rRNA gene sequencing provides foundational insights into the composition and diversity of microbial communities, enabling identification of bacterial taxa associated with MASLD pathogenesis. However, 16S sequencing is limited to taxonomic profiling and lacks resolution of microbial functional potential. Whole metagenome sequencing (WMS) complements this by capturing the entire genetic repertoire of the microbiota, revealing functional genes and metabolic pathways that may be altered in MASLD. This dual approach allows researchers to link specific microbial taxa with their metabolic capabilities, offering a more complete understanding of microbiome-driven mechanisms in disease progression.

Metabolomics, particularly through non-targeted and targeted liquid chromatography–tandem mass spectrometry (LC–MS/MS), quantifies microbial metabolites such as bile acids, short-chain fatty acids (SCFAs), tryptophan derivatives, and trimethylamine N-oxide (TMAO) in fecal and serum samples. These metabolites serve as direct molecular mediators of gut-liver axis communication, influencing hepatic lipid metabolism, inflammation, and fibrosis. For example, bile acid profiles modulated by microbial enzymes can activate nuclear receptors like farnesoid X receptor (FXR), impacting liver homeostasis and MASLD progression. SCFAs, produced by fermentation of dietary fibers, regulate energy balance and immune responses, while tryptophan metabolites modulate intestinal barrier integrity and systemic inflammation. TMAO, derived from microbial metabolism of choline and carnitine, has been implicated in cardiovascular risk associated with MASLD.

Integrative multi-omics analyses enable the construction of association networks linking microbial taxa, functional genes, metabolites, and clinical phenotypes. For instance, in pediatric MASLD, combined 16S rDNA sequencing and targeted metabolomics identified a correlation axis involving the bacterium *Ruminococcus torques*, the secondary bile acid deoxycholic acid (DCA), and liver stiffness measurement (E value), highlighting a mechanistic link between microbial composition, metabolite production, and liver fibrosis severity. Comparable multi-compartment analyses in adults have identified a partly different microbial pattern. In adults with obesity and MASLD, integrated profiling of fecal, hepatic, and adipose tissue microbiomes together with hepatic inflammatory and transcriptomic features showed enrichment of Enterococcus, Granulicatella, and Morganellaceae in MASH, whereas Brevibacterium was enriched in adipose tissue from patients with fibrosis (Schwenger et al., 2025). Thus, pediatric and adult studies both support a relationship between microbial alterations and disease severity, but the specific taxa and functional networks identified are not identical. Age-specific validation should therefore accompany the development of microbiome-derived biomarkers and multi-omics prediction models. Such integrative frameworks facilitate the identification of key microbial-metabolite signatures that may serve as biomarkers or therapeutic targets. Moreover, multi-omics integration supports the elucidation of causal pathways and molecular mediators underlying MASLD pathogenesis, advancing precision medicine approaches tailored to individual microbiome profiles (Zöggeler et al., 2025; Li, Z. et al., 2026; Zhou, H. et al., 2026).

### 6.2 Mendelian randomization and causal inference

Mendelian randomization (MR) leverages genetic variants as instrumental variables to infer causal relationships between exposures, such as gut microbiota features, and disease outcomes, including MASLD. This approach mitigates confounding and reverse causation inherent in observational studies by exploiting the random allocation of alleles at conception. Two-sample MR analyses using large-scale genome-wide association study (GWAS) datasets have identified specific microbial taxa and epigenetic regulators causally linked to MASLD risk.

For example, MR studies have implicated several gut bacterial taxa as causal contributors to MASLD development, while also highlighting the role of m6A RNA methylation regulatory genes in modulating disease susceptibility. Mediation analyses further suggest that gut microbiota partially mediate the association between host genetic variants and MASLD, indicating a gene-microbiome interplay in disease pathogenesis. One notable pathway involves the m6A demethylase ALKBH5, the genus Parabacteroides, and MASLD, forming a potential “m6A-gut microbiome-liver” axis. Although this mediation pathway did not reach statistical significance, it revealed a directional trend suggestive of indirect effects, warranting further investigation. This axis offers a novel mechanistic perspective on how epigenetic modifications and microbiome alterations converge to influence MASLD, opening avenues for targeted interventions that modulate both genetic and microbial factors (Li, Z. et al., 2026).

MR can therefore complement observational microbiome studies by prioritizing candidate causal relationships for downstream experimental testing. Integration with host-genetic data and functional validation is required before MR-derived microbial targets can be incorporated into precision intervention strategies.

### 6.3 Functional validation via fecal microbiota transplantation experiments

Fecal microbiota transplantation (FMT) combined with antibiotic-mediated microbiota depletion serves as a valuable tool for functional validation, supporting causal inference regarding the role of gut microbiota in MASLD pathophysiology. Antibiotic pretreatment substantially depletes the endogenous microbiota, allowing subsequent transplantation of donor fecal material to assess the sufficiency and necessity of specific microbial communities in mediating phenotypic effects.

This experimental design enables rigorous functional validation by demonstrating that protective or pathogenic effects observed in donor animals or humans can be transferred to recipient models. For instance, FMT from animals treated with pharmacological agents or undergoing surgical interventions can reveal whether therapeutic benefits are mediated via microbiota alterations. Studies involving ginseng saponin Rg1, Wuling powder, and Duodenal Jejunal Bypass (DJB) surgery have employed this approach, showing that fecal material from treated donors confers metabolic improvements in recipient mice, thereby supporting a microbiota-mediated component.

Combining FMT with high-throughput microbiome sequencing and metabolite profiling further refines the identification of key functional bacterial taxa and effector metabolites responsible for MASLD modulation. This integrative methodology supports the development of precision microbiome interventions by pinpointing microbial targets and metabolic pathways amenable to therapeutic manipulation. Because most microbiota-transfer experiments are performed in animal recipients, they establish mechanistic plausibility more readily than clinical efficacy. Their translational value is greatest when transfer experiments are combined with metagenomic and metabolomic analyses that identify the microbial organisms, functions, or metabolites responsible for the transferred phenotype (Figure 5) (Groenewegen et al., 2025; Ni et al., 2023; Stols-Gonçalves et al., 2023).

**Figure 5:**
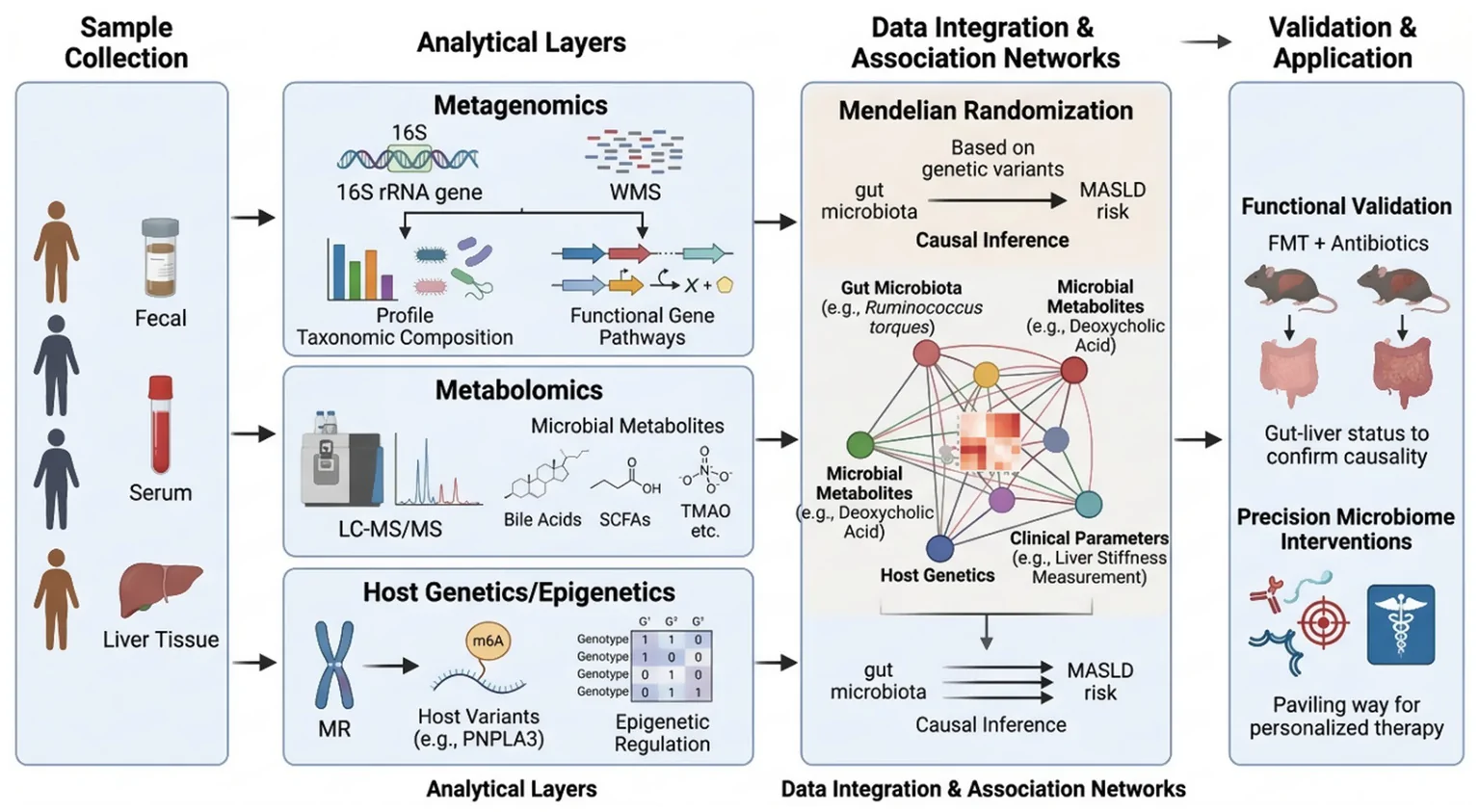
Gut microbiome diversity as a determinant of liver health and disease progression. This comparison illustrates the stark differences between healthy and MASLD livers based on gut microbiome diversity. High microbial diversity is characterized by commensal bacteria enrichment and correlates with a healthy liver. Conversely, reduced microbial diversity, often driven by loss of alpha-diversity and pathogen enrichment (e.g., E. coli), leads to dysbiosis and decreased beneficial bacteria (e.g., Faecalibacterium). This dysbiotic state, potentially predisposed by the PNPLA3 rs738409 genotype, promotes disease progression from simple steatosis to more severe MASH and fibrosis.

## 7 Challenges and future research directions

### 7.1 Limitations and new opportunities in portal vein circulation research

The portal vein serves as the critical conduit through which gut microbiota and their metabolites reach the liver, forming the anatomical and functional basis of the gut-liver axis implicated in metabolic dysfunction-associated steatotic liver disease (MASLD). Despite its central role, current research predominantly relies on indirect assessments of gut-liver interactions by analyzing fecal microbiota composition and peripheral blood metabolites, which limits the mechanistic understanding of MASLD pathogenesis. Direct sampling of portal vein blood would provide more precise insights into the dynamic flux of microbial metabolites and their immediate hepatic effects, yet this approach is hindered by the invasiveness of portal vein catheterization and ethical constraints in human studies. The lack of portal vein-specific metabolomic and microbiome data restricts the ability to delineate causal pathways linking gut dysbiosis to hepatic inflammation, steatosis, and fibrosis. Recent advances in metabolomics and high-throughput sequencing technologies offer promising avenues to characterize portal circulation profiles, potentially uncovering novel biomarkers and therapeutic targets. Moreover, integrating portal vein metabolomics with assessments of hepatic sinusoidal endothelial function could elucidate mechanisms underlying portal hypertension in MASLD, a complication that remains poorly understood. However, the invasiveness and risk associated with portal vein sampling necessitate the development of innovative, minimally invasive diagnostic tools capable of monitoring portal hemodynamics and metabolite dynamics in real time. Such tools would enable longitudinal studies to track disease progression and response to microbiome-targeted therapies, facilitating precision medicine approaches. In summary, overcoming the technical and ethical challenges of portal vein research is essential to deepen mechanistic insights into MASLD and to harness the gut-liver axis for improved diagnosis and treatment strategies (Huang et al., 2025; Lau et al., 2025; Luo et al., 2025).

### 7.2 Individual variability and precision microbiota interventions

The composition and function of the gut microbiota exhibit substantial inter-individual heterogeneity influenced by host genetics, diet, medication history, and lifestyle factors, which significantly impact the efficacy of microbiota-targeted interventions in MASLD. This heterogeneity poses a major challenge to the development of standardized therapeutic strategies, as identical interventions may yield divergent outcomes across patients. For instance, genetic polymorphisms such as PNPLA3 rs738409 modulate lipid metabolism and inflammatory responses, thereby influencing MASLD susceptibility and progression. Stratifying patients based on host genotypes, baseline microbiota profiles, and metabolic phenotypes can enable the design of personalized microbiota modulation regimens, enhancing therapeutic precision and reproducibility. Recent studies employing multi-omics integration and machine learning algorithms have demonstrated the feasibility of constructing predictive models that correlate microbiota features with disease progression and treatment response. These computational approaches can identify microbial signatures predictive of severe steatosis or fibrosis, guiding patient selection and intervention customization. Pharmacomicrobiomic stratification may also be relevant to established metabolic pharmacotherapies. For example, pretreatment fecal microbiome features have been associated with subsequent glycemic response to semaglutide, while microbial metabolic activity has been proposed as a modifier of response to THR-β agonism (Zhang, Y. H. et al., 2025; Klemets et al., 2026). Future MASLD trials should determine whether such baseline microbial signatures predict liver-specific outcomes independently of weight loss and other conventional clinical predictors. Furthermore, machine learning and artificial intelligence-based approaches may facilitate the development of predictive models for selecting probiotic, prebiotic, synbiotic, or fecal microbiota transplantation strategies according to individual microbial ecosystems and metabolic states (Borges-Canha et al., 2025; Li, Z. et al., 2026). Such precision microbiome therapeutics hold promise to overcome the current “one-size-fits-all” paradigm, addressing the complex pathophysiology of MASLD more effectively. However, large-scale, well-controlled clinical trials incorporating stratified patient cohorts and standardized endpoints are required to validate these approaches. Ultimately, integrating host genetics, microbiome data, and clinical parameters into comprehensive predictive frameworks will be pivotal for advancing personalized microbiota-based therapies in MASLD (Huang et al., 2025; Lau et al., 2025; Borges-Canha et al., 2025; Luo et al., 2025).

Importantly, precision microbiome therapy should be operationalized through prospectively defined biological responder phenotypes rather than inferred retrospectively after heterogeneous clinical outcomes have occurred. Future studies should establish a minimum mechanistic framework comprising baseline metagenomic and metabolomic profiling, standardized documentation of habitual diet and microbiome-modifying medications, stratification according to metabolic phenotype and fibrosis stage, longitudinal assessment of microbial target engagement, and prespecified liver-centered clinical endpoints (Lau et al., 2025; Saeed et al., 2025; Li, Z. et al., 2026). Rather than defining a universally “healthy” microbiome, intervention selection may be more informative when based on specific functional deficits, such as impaired short-chain fatty acid production, altered bile acid transformation, or intestinal barrier dysfunction (Lau et al., 2025; Saeed et al., 2025; Li, Z. et al., 2026). For probiotics and synbiotics, this framework should include strain-level characterization and confirmation of functional microbial changes, whereas FMT trials should additionally assess donor–recipient engraftment and determine whether baseline dysbiosis or impaired barrier function identifies patients more likely to respond (Groenewegen et al., 2025; Saeed et al., 2025). Predictive microbial signatures should also undergo external validation across independent and geographically diverse cohorts before being incorporated into patient-selection algorithms (Lau et al., 2025; Borges-Canha et al., 2025; Li, Z. et al., 2026). Such an approach would allow negative trials to distinguish true biological inefficacy from inadequate microbial target engagement or inappropriate patient selection, thereby improving the reproducibility and interpretability of microbiota-directed interventions in MASLD (Saeed et al., 2025; Li, Z. et al., 2026).

### 7.3 Long-term safety and durability of therapeutic effects

While microbiota-targeted therapies such as fecal microbiota transplantation (FMT), probiotics, and prebiotics have shown encouraging short-term benefits in MASLD management, the long-term safety and sustained efficacy of these interventions remain inadequately characterized. Most clinical studies to date have limited follow-up durations, leaving critical questions unanswered regarding the durability of microbiota modulation and potential adverse effects. Safety concerns include the risk of transmitting unknown pathogens through donor microbiota in FMT, horizontal transfer of antibiotic resistance genes, and ecological disturbances leading to dysbiosis or opportunistic infections. Moreover, the dynamic nature of the gut microbiome and host immune responses may result in transient therapeutic effects, necessitating repeated interventions or adjunctive treatments. Establishing robust long-term monitoring frameworks incorporating microbiome sequencing, metabolomic profiling, and clinical assessments is essential to detect delayed adverse events and evaluate sustained benefits. Additionally, standardized protocols for donor screening, microbial preparation, and administration routes must be developed to minimize risks and enhance reproducibility. Another challenge lies in the heterogeneity of MASLD stages and metabolic phenotypes, which complicates the establishment of universal intervention guidelines and efficacy endpoints. International consensus on standardized outcome measures, including histological, biochemical, and patient-reported parameters, is urgently needed to harmonize clinical trials and facilitate regulatory approval. Addressing these challenges will be critical to translating microbiota-based therapies from experimental settings to routine clinical practice, ensuring safe and effective long-term management of MASLD (Huang et al., 2025; Saeed et al., 2025; Luo et al., 2025).

### 7.4 Beyond soluble metabolites: microbial extracellular vesicles and multi-kingdom interactions

An additional limitation of the current gut-liver axis framework is its predominant emphasis on bacterial abundance and freely diffusible microbial metabolites. Gut microorganisms can also communicate with the host through extracellular vesicles (EVs), which represent membrane-enclosed particles capable of transporting biologically active cargo over distances. Bacteria-derived extracellular vesicles (BEVs), including outer membrane vesicles released by Gram-negative bacteria and vesicles produced by Gram-positive species, can contain lipopolysaccharide, peptidoglycan-associated components, proteins, lipids, DNA, RNA, and other signaling molecules (Villard et al., 2021; Qin et al., 2025). Encapsulation within a lipid bilayer may protect these molecules from extracellular degradation and enable their delivery to intestinal epithelial cells or, following barrier translocation, to distant host tissues including the liver (Villard et al., 2021; Qin et al., 2025). Therefore, microbial signaling through the gut-liver axis is not restricted to soluble metabolites or free pathogen-associated molecular patterns.

BEVs may exert either pathogenic or protective effects depending on their microbial origin and molecular cargo. Vesicles containing pro-inflammatory components can activate host pattern-recognition pathways, including TLR4-related signaling, and have been implicated in intestinal barrier dysfunction, insulin resistance, hepatic inflammation, and fibrogenic responses (Villard et al., 2021; Qin et al., 2025). Conversely, vesicles derived from beneficial commensals may reproduce part of the metabolic effects attributed to their parent bacteria. Recent preclinical evidence provides a particularly relevant example in MASH: Romboutsia hominis promoted hepatic lipid accumulation and inflammation, whereas *Akkermansia muciniphila* and its extracellular vesicles attenuated hepatic lipid deposition through regulation of lipid biosynthesis-related pathways (Kwak et al., 2026). These findings suggest that functional microbial products such as EVs may partly explain why taxonomic abundance alone does not consistently predict the metabolic effects of a given microorganism. However, the available MASLD/MASH evidence remains predominantly experimental, and whether circulating gut-derived BEV signatures reliably reflect disease activity or have causal relevance in humans remains uncertain (Villard et al., 2021; Qin et al., 2025; Kwak et al., 2026).

This vesicle-mediated communication should also be considered within a broader multi-kingdom intestinal ecosystem. As discussed above, MASLD-associated alterations extend beyond bacteria to the mycobiome and virome, particularly bacteriophages (Zheng et al., 2025; Lang et al., 2020; Wang, L. et al., 2025; Zhou, X. et al., 2026). Fungi can contribute bioactive metabolites and interact competitively or cooperatively with bacterial communities, whereas bacteriophages can alter bacterial abundance, strain composition, gene transfer, and metabolic capacity (Zheng et al., 2025; Wang, L. et al., 2025; Zhou, X. et al., 2026). Importantly, these components are unlikely to act independently: phage-mediated restructuring of bacterial populations may modify both metabolite production and BEV release, while fungal-bacterial interactions may further reshape the metabolic environment of the gut. Thus, a bacterium-centered analysis based on fecal taxonomic abundance alone may overlook important functional layers of gut-liver communication.

Future studies should therefore adopt a multi-kingdom and multi-modal framework integrating bacterial metagenomics, mycobiome profiling, virome sequencing, metabolomics, and characterization of microbial extracellular vesicles. For BEVs in particular, major methodological challenges include distinguishing microbial vesicles from host-derived EVs in biological samples, establishing standardized isolation and quantification procedures, determining their microbial source and cargo composition, and demonstrating biologically relevant delivery to hepatic cell populations (Villard et al., 2021; Qin et al., 2025). Longitudinal human studies should further determine whether alterations in specific fungal taxa, phage-host networks, or BEV cargo precede MASLD progression rather than simply accompany established metabolic disease. Functional validation using defined microbial strains, purified vesicles, phage-bacteria systems, and appropriate recipient models will be essential before these components can be considered reliable biomarkers or therapeutic targets. Such an expanded framework may provide a more complete representation of gut-liver signaling than the conventional focus on bacterial composition and soluble metabolites alone (Figure 6) (Zheng et al., 2025; Zhou, X. et al., 2026; Villard et al., 2021; Qin et al., 2025).

**Figure 6:**
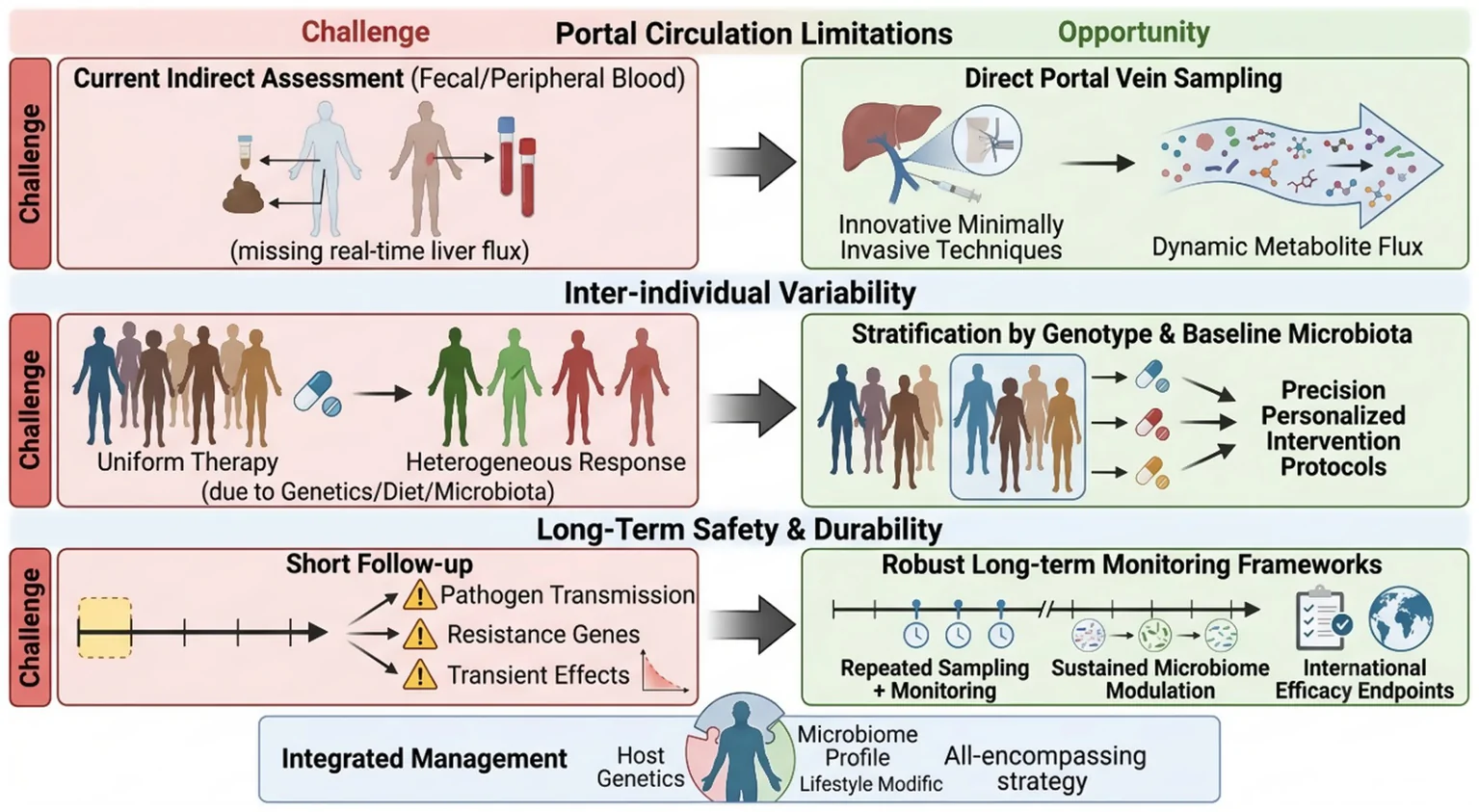
Therapeutic strategies for modulating the gut-liver axis. The illustration showcases various interventions targeting the gut-liver axis to treat MASLD. These include: Probiotics/Prebiotics to increase beneficial bacteria and SCFAs for improved metabolic parameters; Fecal Microbiota Transplantation (FMT) from a healthy donor to restore a healthy microbiota and reduce liver fat content; Natural Products/TCM, such as Ginsenoside Rg1 and herbal formulas, to reshape gut microbiota and influence AHR/IL-22 and Bile Acid metabolism; and Bariatric/Metabolic Surgery to restructure the gut, activate FXR/FGF19 signaling, and improve glucose/lipid metabolism. Novel interventions focus on targeting specific metabolites for precision therapy.

## 8 Conclusion

The intricate involvement of the gut microbiota in the pathogenesis of metabolic dysfunction-associated steatotic liver disease (MASLD) through the gut-liver axis represents a paradigm shift in our understanding of this complex condition. From an expert perspective, the multifaceted dysbiosis characterized by reduced microbial diversity, depletion of beneficial bacteria, and enrichment of pathogenic species underscores the critical role of microbial ecology in modulating hepatic inflammation, steatosis, and fibrosis. This dysregulation manifests through several interconnected mechanisms, including intestinal barrier impairment, bile acid metabolism disturbances, short-chain fatty acid imbalances, aberrant tryptophan metabolism, and oxidative stress regulation. Emerging evidence further expands this framework beyond bacterial taxonomic composition and soluble metabolites. Microbial extracellular vesicles may act as protected carriers of microbial proteins, lipids, nucleic acids, and immunostimulatory molecules, whereas intestinal fungi and bacteriophages add additional layers of cross-kingdom ecological regulation that can reshape microbial metabolism and host signaling. Although these emerging components remain less well characterized than the bacterial metabolome, their integration into future mechanistic and multi-omics studies will be important for achieving a more complete understanding of gut-liver communication in MASLD. These emerging components broaden the conventional bacteria- and metabolite-centered view of gut–liver communication and should be incorporated into future mechanistic and multi-omics investigations of MASLD.

Balancing the diverse research perspectives and findings in this field requires a nuanced appreciation of the complexity inherent in host-microbiome interactions. While preclinical studies robustly demonstrate the causative and modulatory roles of gut microbiota alterations, clinical investigations remain heterogeneous, reflecting variability in study design, patient populations, and intervention modalities. Probiotic, prebiotic, and synbiotic therapies have shown promise in modulating microbial composition and metabolic outputs, yet their efficacy is often inconsistent, likely due to interindividual differences in baseline microbiota and host genetics. Importantly, the maturity of evidence differs substantially across microbiota-targeted interventions. FMT has entered early clinical evaluation but has yielded inconsistent therapeutic outcomes, whereas the microbiome-mediated effects of most TCM-derived bioactive compounds and several metabolite-targeted approaches remain supported predominantly by preclinical studies. Accordingly, mechanistic efficacy in animal models should not be interpreted as evidence of clinical readiness. Although these experimental findings provide valuable proof-of-concept for targeting the gut-liver axis, translation requires rigorous human pharmacological characterization, confirmation of microbial and metabolic target engagement, adequately powered randomized controlled trials with validated liver-centered endpoints, and long-term safety assessment. The microbiota-mediated effects observed following bariatric surgery further emphasize the therapeutic potential of microbiome modulation but also illustrate the complexity of disentangling direct surgical effects from microbial contributions.

A critical challenge lies in reconciling these diverse findings to formulate a coherent therapeutic framework. The interplay between host genetic background, baseline microbial signatures, and environmental influences such as diet and lifestyle creates a highly individualized landscape of microbiota-host interactions. This complexity necessitates a precision medicine approach, tailoring microbiota-targeted therapies to the unique microbial and genetic context of each patient. Such individualized strategies promise to optimize therapeutic efficacy and minimize adverse effects, moving beyond the one-size-fits-all paradigm that has limited progress in MASLD management.

Looking forward, advancing the field demands a concerted focus on several key areas. The development of sophisticated methodologies to directly assess metabolites within the portal circulation and to monitor hepatic hemodynamics will provide critical insights into the dynamic gut-liver metabolic crosstalk. Integrative multi-omics approaches, combining metagenomics, metabolomics, transcriptomics, and proteomics, hold the potential to unravel the complex molecular networks underpinning MASLD and to identify robust biomarkers for disease stratification and therapeutic response prediction. Furthermore, large-scale, long-term clinical trials with well-characterized cohorts are essential to validate microbiota-targeted interventions and to establish standardized protocols for their clinical application.

In conclusion, the evolving landscape of gut microbiota research in MASLD exemplifies the transformative potential of microbiome science in hepatology. By harmonizing mechanistic insights with clinical evidence and embracing the principles of precision medicine, the field is poised to translate microbiota-targeted strategies from bench to bedside effectively. Such advancements will not only enhance our ability to prevent and treat MASLD but also improve the long-term prognosis and quality of life for affected patients, marking a significant milestone in the management of this increasingly prevalent liver disease.
